# Phylogenetically Distant Viruses Use the Same BH3-Only Protein Puma to Trigger Bax/Bak-Dependent Apoptosis of Infected Mouse and Human Cells

**DOI:** 10.1371/journal.pone.0126645

**Published:** 2015-06-01

**Authors:** Emanuela Papaianni, Souhayla El Maadidi, Andrea Schejtman, Simon Neumann, Ulrich Maurer, Francesca Marino-Merlo, Antonio Mastino, Christoph Borner

**Affiliations:** 1 Department of Biological and Environmental Sciences, University of Messina, Via F. Stagno d’Alcontres 31, I-98166, Messina, Italy; 2 The Institute of Translational Pharmacology, CNR, Via Fosso del Cavaliere 100, I-00133, Rome, Italy; 3 Institute of Molecular Medicine and Cell Research, Albert Ludwigs University of Freiburg, Stefan Meier Strasse 17, D-79104, Freiburg, Germany; 4 Faculty of Biology, Albert Ludwigs University of Freiburg, Schänzlestrasse 1, D-79104, Freiburg, Germany; 5 IMBS Program between Albert Ludwigs University of Freiburg, Freiburg, Germany, and University of Buenos Aires, Buenos Aires, Argentina; 6 Spemann Graduate School of Biology and Medicine (SGBM), Albert Ludwigs University of Freiburg, Albertstrasse 19a, D-79104, Freiburg, Germany; 7 BIOSS, Centre for Biological Signaling Studies, Hebelstrasse 2, D-79104, Freiburg, Germany; Roswell Park Cancer Institute, UNITED STATES

## Abstract

Viruses can trigger apoptosis of infected host cells if not counteracted by cellular or viral anti-apoptotic proteins. These protective proteins either inhibit the activation of caspases or they act as Bcl-2 homologs to prevent Bax/Bak-mediated outer mitochondrial membrane permeabilization (MOMP). The exact mechanism by which viruses trigger MOMP has however remained enigmatic. Here we use two distinct types of viruses, a double stranded DNA virus, herpes simplex virus-1 (HSV-1) and a positive sense, single stranded RNA virus, Semliki Forest virus (SFV) to show that the BH3-only protein Puma is the major mediator of virus-induced Bax/Bak activation and MOMP induction. Indeed, when Puma was genetically deleted or downregulated by shRNA, mouse embryonic fibroblasts and IL-3-dependent monocytes as well as human colon carcinoma cells were as resistant to virus-induced apoptosis as their Bax/Bak double deficient counterparts (Bax/Bak-/-). Puma protein expression started to augment after 2 h postinfection with both viruses. Puma mRNA levels increased as well, but this occurred after apoptosis initiation (MOMP) because it was blocked in cells lacking Bax/Bak or overexpressing Bcl-x_L_. Moreover, none of the classical Puma transcription factors such as p53, p73 or p65 NFκB were involved in HSV-1-induced apoptosis. Our data suggest that viruses use a Puma protein-dependent mechanism to trigger MOMP and apoptosis in host cells.

## Introduction

The role of apoptotic, programmed cell death as an ancestral form of host cellular response to inhibit viral replication and limit viral spread and the co-evolutionary capacity of viruses to counteract apoptosis have been extensively investigated in the past years [1–4]. Particularly, large amounts of data have been accumulated on the mechanisms by which viruses subvert the cell death machinery on the mitochondrial level [5–7].

Herpes simplex viruses (HSV) are double-stranded DNA viruses belonging to the *Alphaherpesvirinae* subfamily of herpesviruses. Particularly, herpes simplex virus type 1 (HSV-1) is a human common pathogen that rapidly and efficiently replicates at a portal entry of the host before retrograde transportation to nuclei of sensory neurons. In these cells, HSV-1 remains latent for the lifetime of its host and can be reactivated to cause lesions at or near the initial site of infection. This complex cycle of infection is tightly controlled by an ordered sequence of molecular events, involving a regulated expression of both viral and cellular genes [8]. As a consequence, it is not surprising that HSV-1, similarly to other viruses, was found to block apoptosis at multiple stages of infection to prevent the host cell from dying prematurely [9,10]. Several HSV-1 proteins are involved in counteracting apoptosis. They include the immediate-early proteins ICP4 [11], ICP27 [12] and ICP22 [13], the late protein kinase US3 [13–16], the late viral glycoproteins gD and gJ [17–20], and the latency associated transcript (LAT) [21]. On the cellular side NFκB [18,22,23] and members of the Bcl-2 family [6,7,24] seem to play the most important roles in protecting HSV-infected cells from apoptosis. In particular, the envelope protein gD of HSV-1 triggers a signalling cascade in infected host cells that leads to the activation of NFκB and inhibition of apoptosis [18,23]. NFκB is known to regulate the expression of a variety of anti-apoptotic genes. Interestingly, we found that HSV-1 induces the up-regulation of the survival proteins FLIP, c-IAP2 and survivin in an NFκB-dependent manner [18]. Thus, depending on the abundance and/or activity of NFκB and its target gene products, cells can be more or less susceptible to HSV-1 induced apoptosis. Moreover, we previously showed that in U937 monocytic cells infected with HSV-2 Bcl-2 overexpression caused increased resistance to virus-induced apoptosis and higher virus yields indicating in a direct manner that manipulation of apoptotic pathways can influence the efficiency of HSV replication at least in certain cell types [24]. However, we have not yet identified the apoptotic component, activated by the virus, which is the target for Bcl-2-mediated cytoprotection.

In fact, depending on a variety of both viral and cellular factors, host cells can also die after HSV-1 infection. For example, whilst apoptosis is usually prevented by wild type HSV-1 in fully permissive epithelial cells, the same virus and the closely related herpes simplex virus type-2 (HSV-2) can induce apoptosis as an exclusive cytopathic effect in human monocytic cells [25]. Induction of apoptosis following HSV-1 infection has also been shown in T lymphocytes [26] and dendritic cells [27,28]. HSV-1 infection resulted in apoptosis of neuronal cells constituting the majority of cells in rat hippocampal cultures [29]. Moreover, recent results suggest that apoptosis may facilitate the exit of HSV-1 from latency [30].

Other viruses such as the positive sense, single stranded RNA virus Semliki Forest (SFV) of the genus *Alphaviridae* do not carry any survival factors in their genome and induce apoptosis of many different mammalian host cell types [31]. The main targets of SFV are immature neurons, which succumb to SFV-induced apoptosis during the first 21 days after birth. Later the virus is apathogenic due to efficient host immune responses although some animals can develop encephalopathies after SFV infection. We previously reported that SFV induces a Bak-dependent apoptosis signalling pathway which can be partially blocked by Bcl-2 overexpression [32]. Recently, we extended our studies to show that in addition to this classical pathway, dsRNA produced during the replication cycle of SFV also triggers a Bax/Bak-independent signalling pathway via the innate immunity sensor MAVS, which recruits caspase-8 on mitochondria and activates caspase-3 in a death receptor-independent manner [33]. While this study provided a link between the virus (dsRNA) and the apoptotic host machinery (MAVS, caspase-8/caspase-3) for the Bax/Bak-independent pathway, we still do not know how Bax/Bak are activated by SFV.

Activation of Bax/Bak leads to mitochondrial outer membrane permeabilization (MOMP), which is an essential step for the release of apoptogenic factors such as cytochrome c to activate effectors caspase-3 and -7 along the intrinsic, mitochondrial signalling pathway of apoptosis [34]. This activation requires the action of a subgroup of the Bcl-2 family, called the BH3-only proteins, which either directly activate Bax/Bak (such as tBid, Bim or Puma) or bind to Bcl-2-like survival factors to release from them prebound Bax/Bak for oligomerization and MOMP induction [35]. BH3-only proteins act as apoptotic sentinels in this process and can be engaged by apoptotic stimuli either by transcriptional induction (Bim, Puma, Noxa, Bmf), posttranslational phosphorylation (Bim, Puma, Bad) or proteolytic cleavage (Bid) [36]. Which BH3-only protein(s) is/are activated by viruses has remained largely unknown. Fischer et al. recently reported that an Ankara vaccinia virus variant lacking the functional Bcl-2 homolog F1L requires Noxa to induce apoptosis of host cells although the molecular mechanism of Noxa activation was not identified [37]. On the other hand, many viruses such as adeno- (E1B-55K), hepatitis (HBx) and papillomaviruses (E6/E7) target the transcription factor p53, which is known to induce Puma or Noxa for apoptosis induction [6]. Consistent with this notion, measles virus has recently been proposed to prevent host cell apoptosis via inhibiting Puma induction by p73, a p53 homolog [38].

In addition to the intrinsic mitochondrial pathways some viruses also induce apoptosis via the extrinsic death receptor pathway. Here caspase-8 is the major target as viruses produce factors, which inhibit this caspase (cowpox crmA, v-FLIP, IAPs) [6]. Caspase-8 is recruited as a cytosolic monomeric enzyme to activated Fas, TNF-R1 or TRAIL death receptors via the adapter molecule FADD [39]. In this so called death-inducing signalling complex (DISC) caspase-8 is activated by proximity-mediated dimerization and then either directly cleaves and activates caspase-3 leading to mitochondria-independent apoptosis (so called type I pathway) or first cleaves Bid whose product tBid then migrates to mitochondria to activate Bax/Bak and MOMP (type II pathway) [39]. While we found that caspase-8 is indeed important for SFV-induced apoptosis in a Bax/Bak-independent manner, this does not involve death receptors but mitochondrial MAVS, forming a new non-canonical DISC [33]. Although it was previously reported that gD and gJ or HSV-1 can protect cells against FasL-induced apoptosis [17,18] and that HSV-1 induces apoptosis of dendritic cells by downregulating c-FLIP [28], it is not known how important the death receptor signalling is for HSV-induced apoptosis.

Here we show that both HSV-1 and SFV primarily use Bax/Bak-dependent, death receptor-independent signalling to induce apoptosis in human monocytes and colon carcinoma cells as well as in mouse embryo fibroblasts and monocytes. The BH3 sensor was uncovered to be Puma for both viruses. Although Puma mRNA was consistently upregulated in response to both HSV-1 and SFV, this effect was dependent on Bax/Bak indicating that it occurred after apoptosis induction. However, Puma protein levels were increased early after HSV-1 and SFV infection by a so far unknown mechanism indicating that this posttranslational regulation is most likely the way by which both viruses trigger Bax/Bak-mediated MOMP and apoptosis if not counteracted by Bcl-2-like survival factors.

## Results

### HSV-1 induces apoptosis in a Bax/Bak-dependent manner in human and mouse cells after viral reproduction

**Fig 1:**
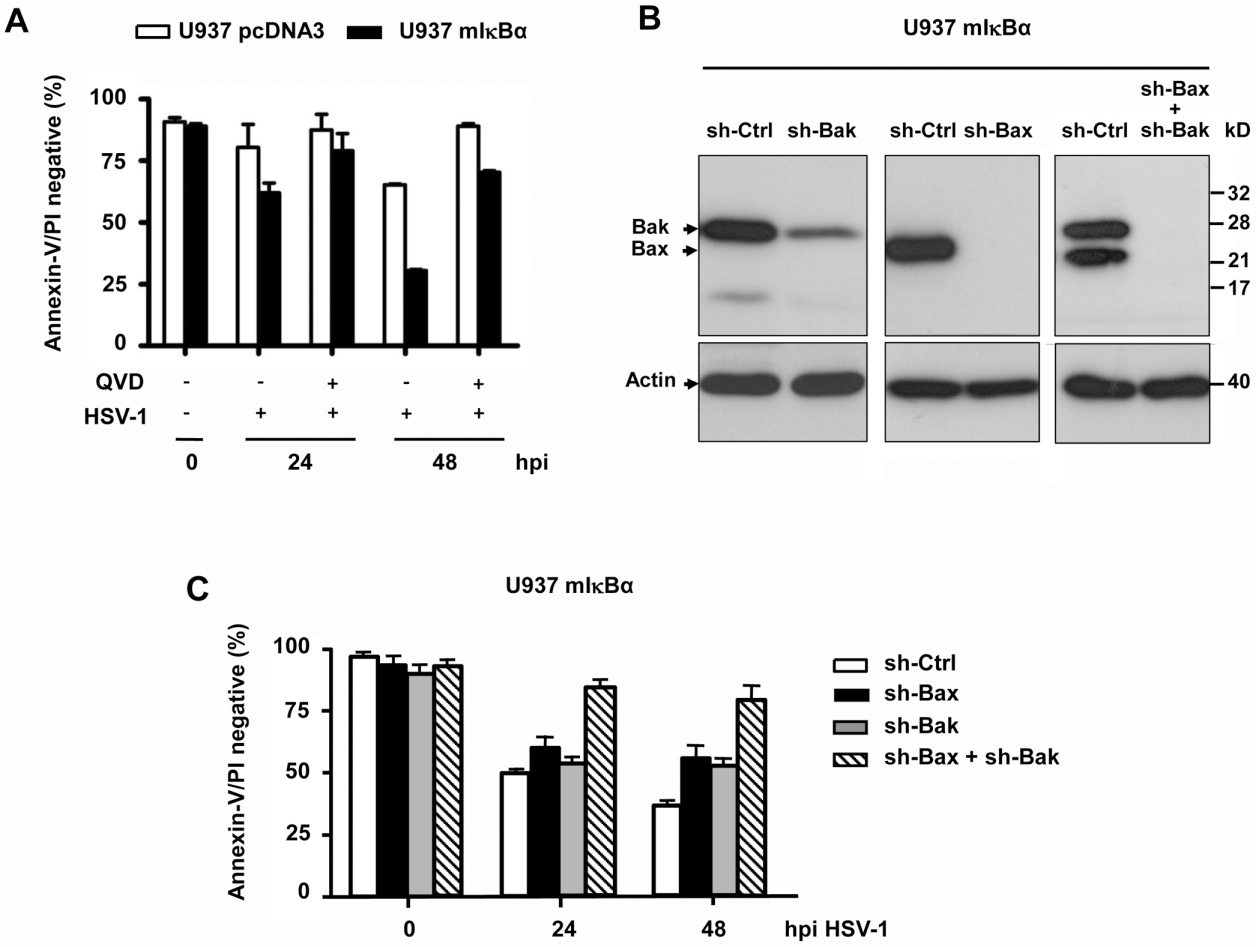
HSV-1-induced apoptosis of U937 monocytes depends on Bax/Bak and is most efficient when NFκB activation if prevented. **(A)** Annexin-V/PI FACS analysis of human U937 monocytes carrying the pcDNA3 vector or expressing a dominant-negative version of IκBα (mIκBα) (which prevents NFκB activation), infected with 50 moi of herpes simplex virus-1 (HSV-1) in the presence or absence of 25 μM of the general caspase inhibitor QVD for 0, 24 and 48 h. The number of cells lacking annexin-V/PI staining (the lower left quadrants in S1 Fig) are depicted. They represent cells which are protected against both apoptotic and necroptosis/necrotic cell death. **(B)** Anti-Bax and anti-Bak western blot analysis of total extracts of puromycin-selected, mixed population U937 mIκBα cells infected with lentivirus carrying a scrambled shRNA (sh-Ctrl) or shRNAs of human Bax (sh-Bax), Bak (sh-Bak) or both. Anti-actin as loading control. **(C)** Annexin-V/PI FACS analysis of the cell lines described in (B) after infecting them with 50 moi of HSV-1 for 0, 24 or 48 h. Data in (A) and (C) are the means of at least three independent experiments ± SEM. The *p* values are the following: (A) mIκBα versus pcDNA3: *p* = 0.008 for 24 h, *p* = 0.003 for 48 h; mIκBα + QVD versus mIκBα - QVD: *p* = 0.01 for 24 h, *p* = 0.005 for 48 h, n = 4. (C) sh-Bax + sh-Bak versus sh-Ctrl, *p* < 0.001 for 24 and 48 h; sh-Bax or sh-Bak versus sh-Ctrl, not significant, n = 5. hpi: hours post infection. kD: kilo Dalton.

HSV-1 only minorly induces apoptosis of permissive human cells, probably because the viral proteins ICP4 and US3 block caspase-dependent and-independent cell death [11,13]. However, some cell types, including human monocytes, show appreciable or even high sensitivity to HSV-1-induced apoptosis [25]. In this case Bcl-2 overexpression can completely block this cell death [24]. Activation of the survival factor NFκB as a consequence of HSV-1 gD/HVEM receptor interaction protects human monocytic U937 cells from apoptosis [23]. We therefore inhibited NFκB activation by expressing a non-phosphorylatable IκBα variant in these cells (U937 mIκBα) and subsequently infected them with 50 moi of HSV-1. While up to 60% of the vector control U937 pcDNA3 cells survived the HSV-1 infection after 48 h, only 25% of the U937 mIκBα cells were still alive at this time point, as assessed by their lack of annexin-V/PI FACS staining (which quantitatively measures cells protected against both apoptosis and secondary necrosis, see lower left quadrants in the dot plots of S1 Fig) (Fig 1A). To elucidate if HSV-1-induced apoptosis was mediated via the intrinsic, mitochondrial signalling pathway in these cells we treated them with the general caspase inhibitor QVD (Fig 1A). Besides, downregulation of Bax and/or Bak was performed with lentiviral-mediated transduction of respective shRNAs (Fig 1B). As shown in Fig 1C, U937 mIκBα cells depleted of Bax and/or Bak were significantly protected from HSV-1-induced apoptosis after 24 and 48 h as compared to those expressing a scrambled control shRNA. A similar extent of protection was achieved in U937 mIκBα cells treated with QVD (Fig 1A, S1 Fig). These data indicate that HSV-1 induces effective apoptosis of human monocytes via the intrinsic Bax/Bak- and caspase-dependent pathway if NFκB activation is ablated.

**Fig 2:**
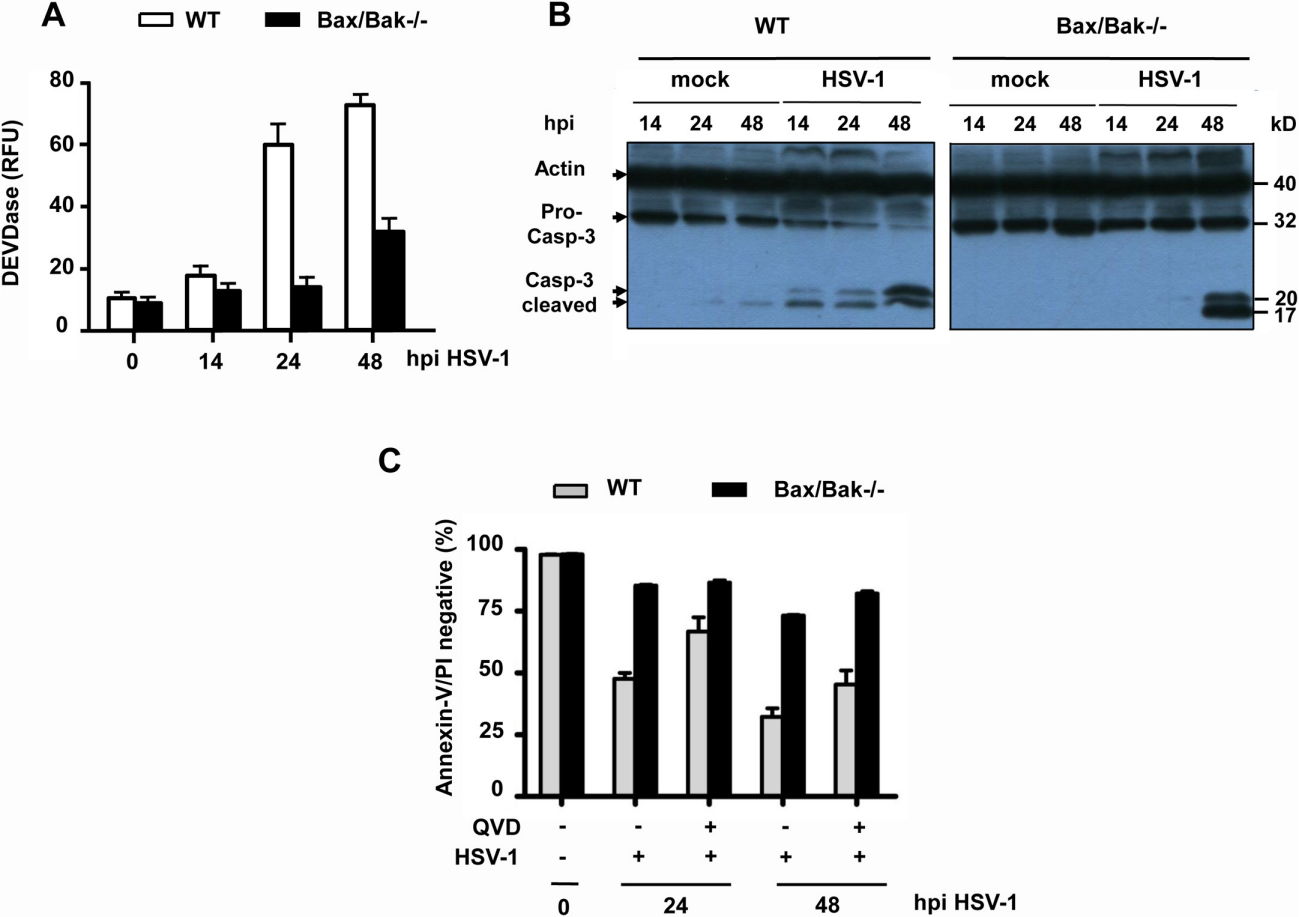
HSV-1-induced caspase-3 activation and apoptosis of SV40 TAg MEFs are predominantly mediated via Bax/Bak. **(A)** Caspase-3/-7 activity (DEVDase) assay and **(B)** anti-caspase-3 (pro-caspase-3 and cleaved caspase-3) western blots of total extracts as well as **(C)** annexin-V/PI FACS analysis of SV40 TAg WT and Bax/Bak-/- MEFs infected with 10 moi of HSV-1 for 0 (mock), 14, 24 or 48 h (hpi) in the absence or presence of 25 μM QVD. The number of cells lacking annexin-V/PI staining (the lower left quadrants in S1 Fig) are depicted in (C). Anti-actin as loading control in (B). Data in (A) and (C) are the means of at least three independent experiments using three different clones of WT and Bax/Bak-/- cells ± SEM. The *p* values are the following: (A) Bax/Bak-/- versus WT cells: *p* < 0.001 for 24 and 48 h, n = 5. (C) Bax/Bak-/- versus WT cells: *p* < 0.001 for 24 and 48 h; WT + QVD versus WT—QVD: *p* = 0.005 for 24 h, *p* = 0.01 for 48 h; Bax/Bak-/- + QVD versus Bax/Bak-/-—QVD: *p* = 0.05 for 24 h, *p* = 0.03 for 48 h, n = 5.

**Fig 3:**
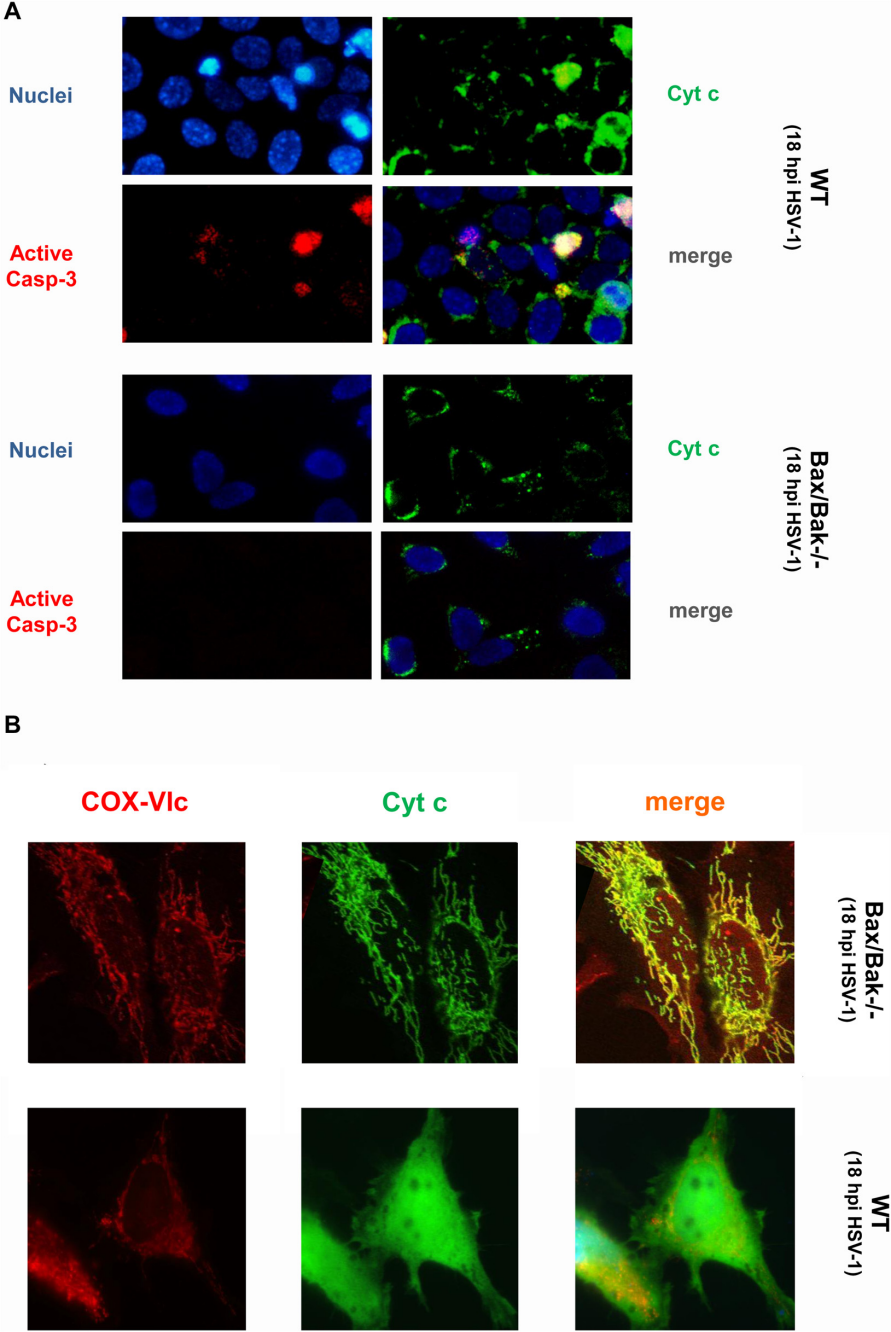
HSV-1-induced cytochrome c release is mediated via Bax/Bak. **(A)** Anti-cytochrome c, anti-active caspase-3 and Hoechst 33334 (nuclei) and **(B)** anti-COX-VIc (mitochondrial marker) and anti-cytochrome c immunofluorescence analyses of SV40 TAg WT and Bax/Bak-/- MEFs infected with 10 moi of HSV-1 for 18 h (hpi). Magnifications in (A) and (B) are 400 and 1000 fold, respectively.

To further characterize HSV-1-induced apoptosis in a genetically more amenable system, we used mouse embryo fibroblasts (MEFs), either transformed by SV40 T antigen (TAg) or spontaneously immortalized (3T9). Following infection with 10 moi of HSV-1, a high percentage of SV40 TAg WT MEFs stained positive for the env protein gD (S2 Fig) indicating that mouse fibroblasts were successfully infected with HSV-1. Concomitantly, HSV-1 triggered in these cells a significant increase in cytosolic caspase-3 activity (DEVDase) (Fig 2A), caspase-3 processing to the active p17 form (Fig 2B) and apoptosis induction (Fig 2C, S1 Fig). Apoptosis was markedly delayed by QVD treatment during the HSV-1 infection (Fig 2C, S1 Fig) although not as much as in U937 cells (compare to Fig 1A, S1 Fig). Moreover, as shown in Fig 3A and 3B, HSV-1-infected cells exhibited a diffuse staining of cytochrome c indicative of its release from punctate and/or elongated mitochondrial structures, a positive staining with active caspase-3 antibodies and nuclear condensation/fragmentation. These data suggest that HSV-1 triggers effective caspase-dependent and-independent apoptosis of SV40 TAg WT MEFs via the intrinsic mitochondrial pathway.

Indeed, when we infected SV40 TAg Bax/Bak-/- MEFs with HSV-1, these cells did not show any cytochrome c release (Fig 3A and 3B), active caspase-3 in the cytoplasm (Figs 2A and 3A) or caspase-3 processing (Fig 2B) for the first 24 h. Moreover, they were largely protected from apoptosis as demonstrated by the virtual lack of annexin-V/PI FACS staining (Fig 2C, S1 Fig) and nuclear condensation/fragmentation (Fig 3A). Only after 48 h, Bax/Bax-deficient MEFs revealed caspase-3 activation/processing (Fig 2A and 2B) and apoptosis (Fig 2C, S1 Fig) indicating that HSV-1 also induced a Bax/Bak-independent, but still caspase-dependent apoptosis signalling pathway as we have recently reported for Semliki Forest Virus (SFV) [33]. Consistent with this notion pre-treatment of Bax/Bak-/- cells with QVD enhanced their protection from apoptosis at 48 h postinfection (Fig 2C, S1 Fig). As previously reported for Bcl-2 overexpression in U937 cells [24], Bax/Bak-/- MEFs exhibited a higher infection rate and therefore increased gD staining as compared to their WT counterparts (S2 Fig). Especially after 72 h postinfection, more gD-positive Bax/Bak-/- than WT cells were counted because the former cells survive longer (S2 Fig). Similarly, HSV-1 viral titers were slightly higher after 48–72 h postinfection when Bax and Bak were depleted in MEFs or Bcl-2 was overexpressed in U937 monocytes (S2 Fig). Importantly, however, both U937 and MEF WT cells still produced high viral titers during early phases of infection (0–48 h) (S2 Fig), indicating that HSV-1 replication and progeny formation occurred before host cell apoptosis induction as previously shown for SFV [32,33].

**Fig 4:**
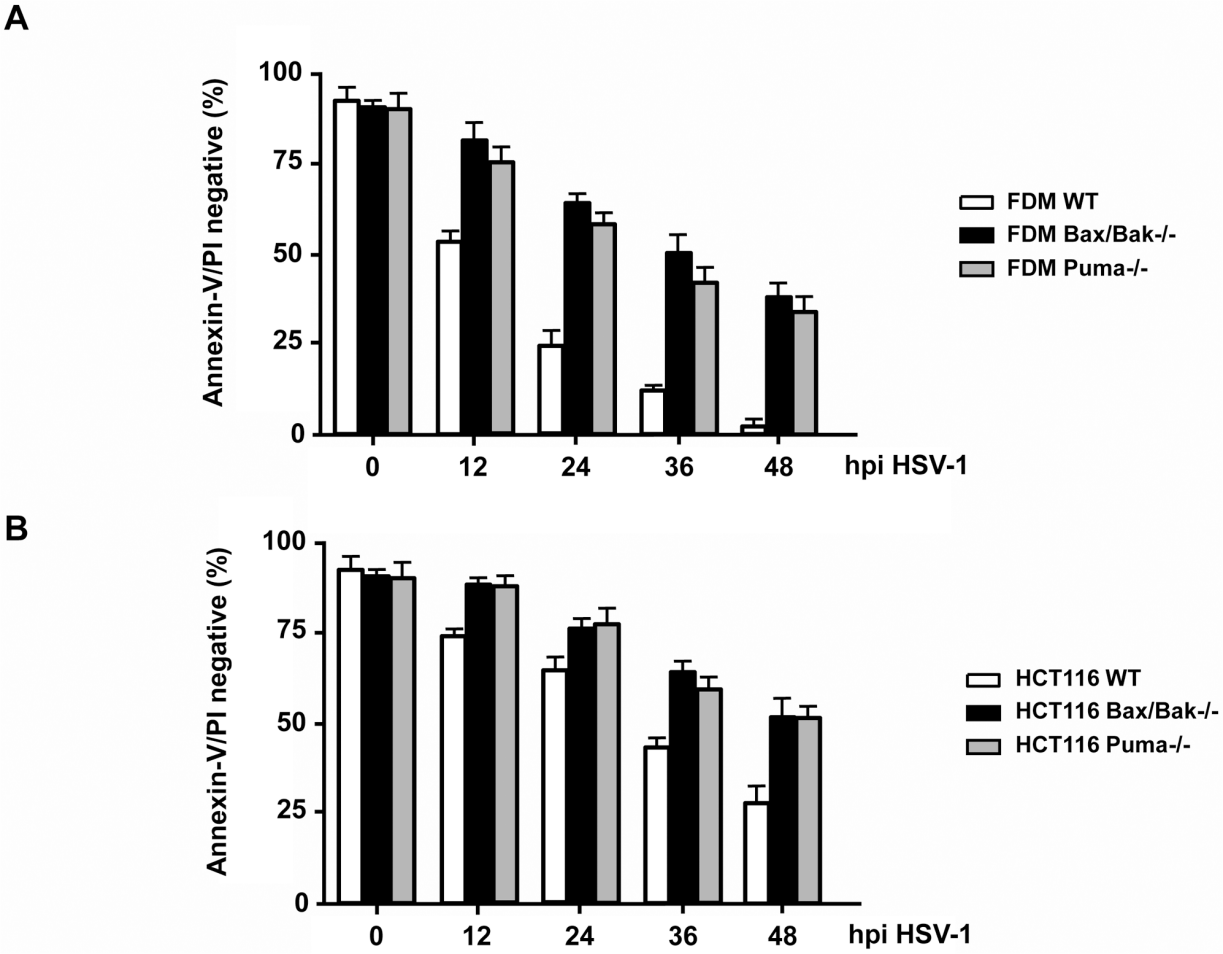
HSV-1 also induces apoptosis of factor-dependent mouse monocytes (FDM) and human carcinoma cells (HCT116), dependent on Bax/Bak and Puma. **(A)** Annexin-V/PI FACS analysis of WT, Puma-/- and Bax/Bak-/- FDMs and **(B)** of WT, Puma-/- and Bax/Bak-/- HCT116 cells infected with 10 moi of HSV-1 for 0, 12, 24, 36 or 48 h (hpi). The number of cells lacking annexin-V/PI staining (the lower left quadrants in S1 Fig) are depicted. Data are the means of at least three independent experiments using three different clones of WT, Puma-/- and Bax/Bak-/- cells in (A) and one clone of each genotype in (B) ± SEM. The *p* values are < 0.001 for Bax/Bak-/- versus WT and Puma-/- versus WT cells for all time points in both (A) and (B), n = 5.

We confirmed the Bax/Bak requirement for HSV-1-induced apoptosis in other mouse cells, IL-3 (factor)-dependent monocytes (FDMs) as well as in human HCT116 colon carcinoma cells. As shown in Fig 4A and 4B and S1 Fig, FDMs and HCT116 cells lacking Bax and Bak expression consistently displayed a higher number of annexin-V/PI negative, surviving cells than their respective WT counterparts at any time postinfection with HSV-1. Like U937 monocytes (Fig 1A), HCT116 cells (Fig 4B, S1 Fig) were not as sensitive to HSV-1-induced apoptosis as mouse fibroblasts (Fig 2C, S1 Fig) and monocytes (Fig 4A, S1 Fig) supporting the notion that human cells can be killed by HSV-1 in a Bax/Bak-dependent, Bcl-2-inhibitable manner, but some survival pathway (most likely mediated by NκFB, see Fig 1) counteracts this process.

### HSV-1 does neither use FasL, TNFα, TRAIL signalling nor RIP1- or RIP3-mediated necroptosis to kill target cells

**Fig 5:**
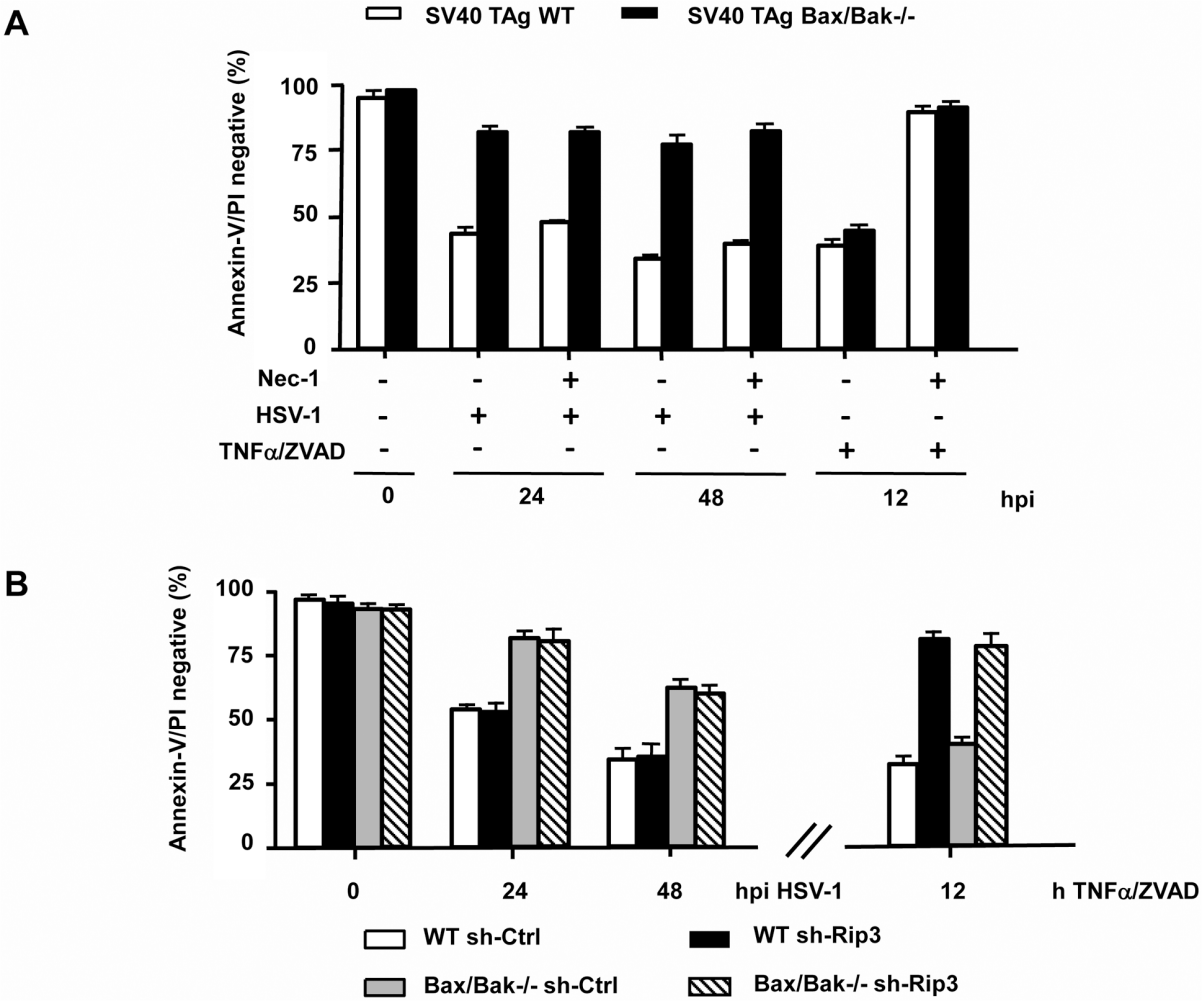
HSV-1-induced cell death does not involve RIP1- and/or RIP3-mediated necroptosis. **(A)** Annexin-V/PI FACS analysis of SV40 TAg WT and Bax/Bak-/- MEFs infected with 10 moi of HSV-1 for 0, 24 or 48 h (hpi) or treated with 10 ng/ml TNFα/100μ M ZVAD-fmk ± 100 μM Necrostatin-1 (Nec-1) for 12 h. **(B)** Annexin-V/PI FACS analysis of mixed populations of SV40 TAg WT and Bax/Bak-/- MEFs stably expressing either sh-Ctrl or sh-Rip3, infected with 10 moi of HSV-1 for 0, 24 or 48 h (hpi) or treated with 10 ng/ml TNFα/100 μM ZVAD-fmk for 12 h. Data are the means of at least three independent experiments ± SEM. The *p* values are the following: (A) HSV-1-infected Bax/Bak-/- versus WT cells: *p* < 0.001 for 24 and 48 hpi; TNFα/ZVAD + Nec-1 versus TNFα/ZVAD—Nec-1 for both WT and Bax/Bak-/- cells: *p* < 0.001; HSV-1 + Nec-1 versus HSV-1—Nec-1 for both WT and Bax/Bak-/- cells: not significant, n = 4. (B) HSV-1-infected Bax/Bak-/- sh-Ctrl versus WT sh-Ctrl and Bax/Bak-/- sh-Rip3 versus WT sh-Rip3: *p* < 0.001 for 24 and 48 hpi; HSV-1-infected Bax/Bak-/- sh-Ctrl versus Bax/Bak-/- sh-Rip3 and HSV-1-infected WT sh-Ctrl versus WT sh-Rip3: not significant, n = 4. TNFα/ZVAD-treated WT sh-Rip3 versus WT sh-Ctrl and TNFα/ZVAD-treated Bax/Bak-/- sh-Rip3 versus Bax/Bak-/- sh-Ctrl: *p* < 0.001, n = 3.

**Fig 6:**
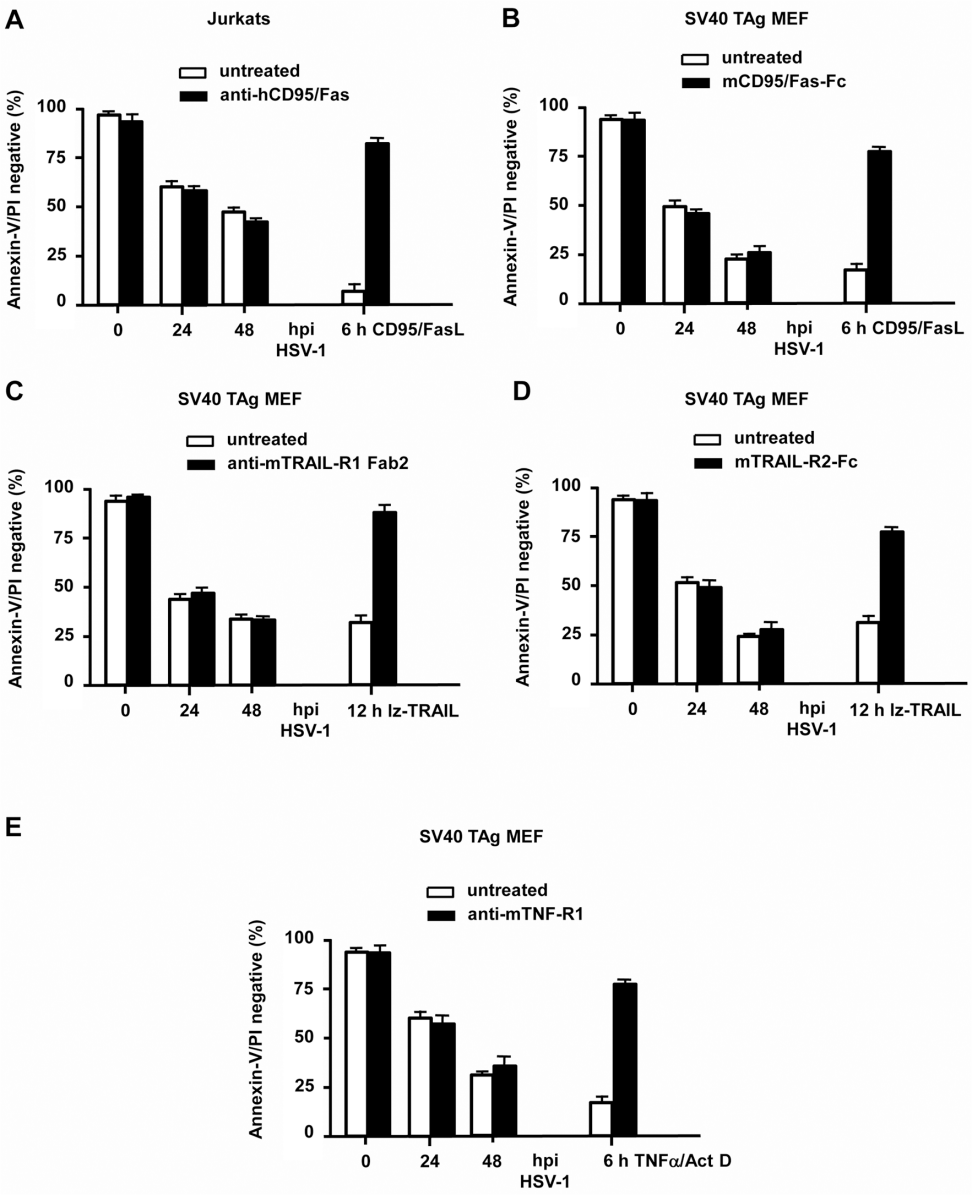
HSV-1-induced apoptosis does not require CD95/Fas, TNFα or TRAIL signalling. Annexin-V/PI FACS analysis of **(A)** human Jurkat cells or **(B-E)** SV40 TAg MEFs infected with 10 moi of HSV-1 for 0, 24 or 48 h in the absence and presence of (A) 10 μg/ml neutralizing anti-hCD95/Fas, (B) 5 μg/ml recombinant mCD95/Fas-Fc protein (capturing the mFasL), (C) 50 μg/ml neutralizing anti-mTRAIL-R1 Fab2, (D) 5 μg/ml recombinant mTRAIL-R2-Fc protein (capturing mTRAIL) or (E) 5 μg/ml neutralizing anti-mTNF-R1. Instead of HSV-1 infections, the following treatments are shown as positive controls: (A, B) 50 ng/ml CD95/FasL for 6 h, (C, D) 500 ng/ml leucine-zipper TRAIL (lz-TRAIL) for 12 h, (E) 10 ng/ml TNFα/1 μM Act D for 6 h ± the respective neutralizing antibody or inhibitor recombinant protein. Data are the means of at least three independent experiments ± SEM. The *p* values are the following: FasL, lz-TRAIL and TNFα/Act D + antibody or recombinant protein versus untreated: *p* < 0.001 for all. HSV-1 + antibodies or recombinant proteins versus untreated: not significant, n = 4.

Since Bax/Bak-/- MEFs still died in a protracted manner by both caspase-dependent and-independent mechanisms, we envisaged the possibility that HSV-1 could also either engage the extrinsic death receptor and/or the necroptotic signalling pathway(s). Necroptosis can be induced by cellular treatment with TNFα + ZVAD and is mediated by RIP1 and RIP3 kinases [40]. To study the involvement of both protein kinases, we knocked-down RIP3 by shRNA (S3 Fig) and inhibited RIP1 using the selective inhibitor necrostatin-1 (Nec-1) (Fig 5A). Both SV40 TAg-transformed WT and Bax/Bak-/- MEFs were effectively killed with TNFα + ZVAD and this cell death was blocked by Nec-1 treatment (Fig 5A) or RIP3 downregulation (Fig 5B). However, neither Nec-1 (Fig 5A) nor the absence of RIP3 expression (Fig 5B) were able to delay or inhibit HSV-1-induced cell death of SV40 TAg WT or Bax/Bak-/- MEFs at any time postinfection indicating that HSV-1 does not induce necroptosis. To test the role of FasL, TNFα or TRAIL in HSV-1-induced apoptosis, we treated Jurkat cells and SV40 TAg MEFs with neutralizing antibodies or recombinant Fc proteins against FasL, Fas, TRAIL, TRAIL-R1 or TNF-R1 before and during HSV-1 infection. As shown in Fig 6A–6E, while the neutralizing agents prevented apoptosis induced by the respective ligands (positive control), they were incapable of protecting either Jurkats or MEFs from HSV-1-induced apoptosis indicating that HSV-1 does not induce and/or use FasL, TNFα or TRAIL or their respective receptors to kill infected cells by apoptosis.

### HSV-1 primarily depends on the BH3-only protein Puma for apoptosis induction

**Fig 7:**
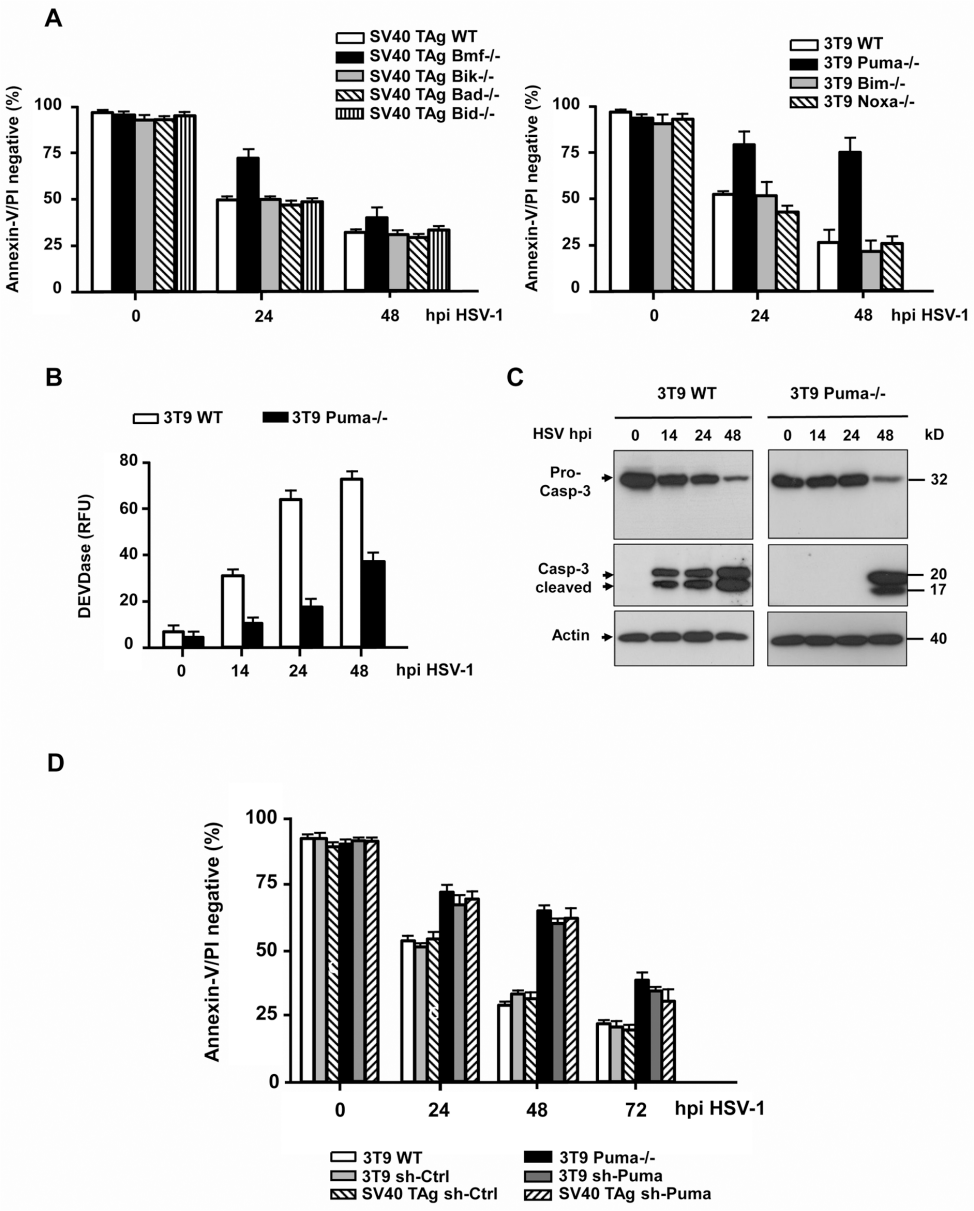
HSV-1-induced caspase-3 activation and apoptosis requires Puma and to a smaller extent Bmf. **(A)** Annexin-V/PI FACS analysis of SV40 TAg WT, Bmf-/-, Bik-/-, Bad-/-, Bid-/- and 3T9-immortalized WT, Puma-/-, Bim-/- and Noxa-/- MEFs infected with 10 moi HSV-1 for 0, 24 or 48 h (hpi). **(B)** Caspase-3/-7 (DEVDase) activity assay and **(C)** anti-caspase-3 (pro-caspase-3 and cleaved caspase-3) western blot analysis of total extracts of 3T9 WT and Puma-/- MEFs infected with HSV-1 for 0, 14, 24 or 48 h. Anti-actin as loading control in (C). **(D)** Annexin-V/PI FACS analysis of puromycin selected, mixed populations of SV40 TAg-transformed and 3T9-immortalized MEFs infected with lentiviruses carrying either a scrambled shRNA (sh-Ctrl) or a shRNA of mouse Puma (sh-Puma), infected with HSV-1 for 0, 24, 48 or 72 h (hpi). The 3T9 WT and Puma-/- cells are shown as controls. Data in (A), (B) and (D) are the means of at least three independent experiments using two different clones of WT and each knock-out cell line in (A) and (B) and mixed populations in (D) ± SEM. The *p* values are the following: (A) Bmf-/- versus WT: *p* = 0.005 for 24 h, *p* = 0.01 for 48 h; Puma-/- versus WT: *p* < 0.001 for 24 h and 48 h, n = 6. (B) Puma-/- versus WT: *p* < 0.001 for 14, 24 h and 48 h, n = 5. (D) sh-Puma versus sh-Ctrl and Puma-/- versus WT: *p* < 0.001 for 24 h, 48 h and 72 h, n = 4.

The activation of Bax/Bak depends on the transcriptional induction or posttranslational modification of upstream BH3-only proteins. To uncover which BH3-only protein was engaged by HSV-1 we infected various 3T9-immortalized or SV40 TAg-transformed MEFs deficient for particular BH3-only proteins with 10 moi of HSV-1 and tested their apoptosis sensitivity. SV40 TAg MEFs lacking Bid, Bad or Bik or 3T9 MEFs lacking Bim or Noxa died in a similar fashion in response to HSV-1 infection as the respective WT cell lines (Fig 7A). SV40 TAg Bmf-/- MEFs were partially protected from apoptosis after 24 h but remained sensitive at 48 h postinfection. The best death protection was achieved in 3T9 MEFs lacking Puma. Here after 24 and 48 h, the cells were as resistant to HSV-1-induced apoptosis as Bax/Bak-/- cells (compare Figs 7A and 2C). This was confirmed in Puma-/- FDMs and HCT116 cells, which survived a HSV-1 infection in a similar way as their Bax/Bak-/- counterparts (Fig 4A and 4B, S1 Fig). The cell death protection was also observed when measuring caspase-3 activation. As with Bax/Bak-/- cells, Puma-/- MEFs did not exhibit any major cytosolic caspase-3 activity or caspase-3 processing at 24 h but only at 48 h postinfection (Fig 7B and 7C).

To assure that the protection from HSV-1-induced apoptosis in Puma-/- cells was not due to another cell death resistance mechanism that had been acquired through the generation of Puma-/- mice or the culturing and/or immortalization of isolated Puma-/- MEFs, we knocked-down Puma expression in both 3T9 and SV40 TAg MEFs by shRNA. As shown in S4 Fig, especially in the SV40 TAg MEFs we did not succeed to fully ablate Puma expression after lentiviral transfer of Puma shRNA as compared to respective scrambled shRNA controls. Nevertheless, both 3T9 and SV40 TAg Puma knock-down MEFs were significantly protected from HSV-1-induced apoptosis up to 72 h postinfection as compared to the respective 3T9 and SV40 TAg control cells (Fig 7D). These data clearly show that Puma is the primary BH3-only protein mediating Bax/Bak and caspase-3 activation and apoptosis in response to HSV-1 infection in both mouse and human cells.

### Both Puma mRNA and protein levels are increased by HSV-1, but transcriptional induction of Puma occurs downstream of MOMP and neither p53, p73 nor p65 NFκB are involved in apoptosis induction

**Fig 8:**
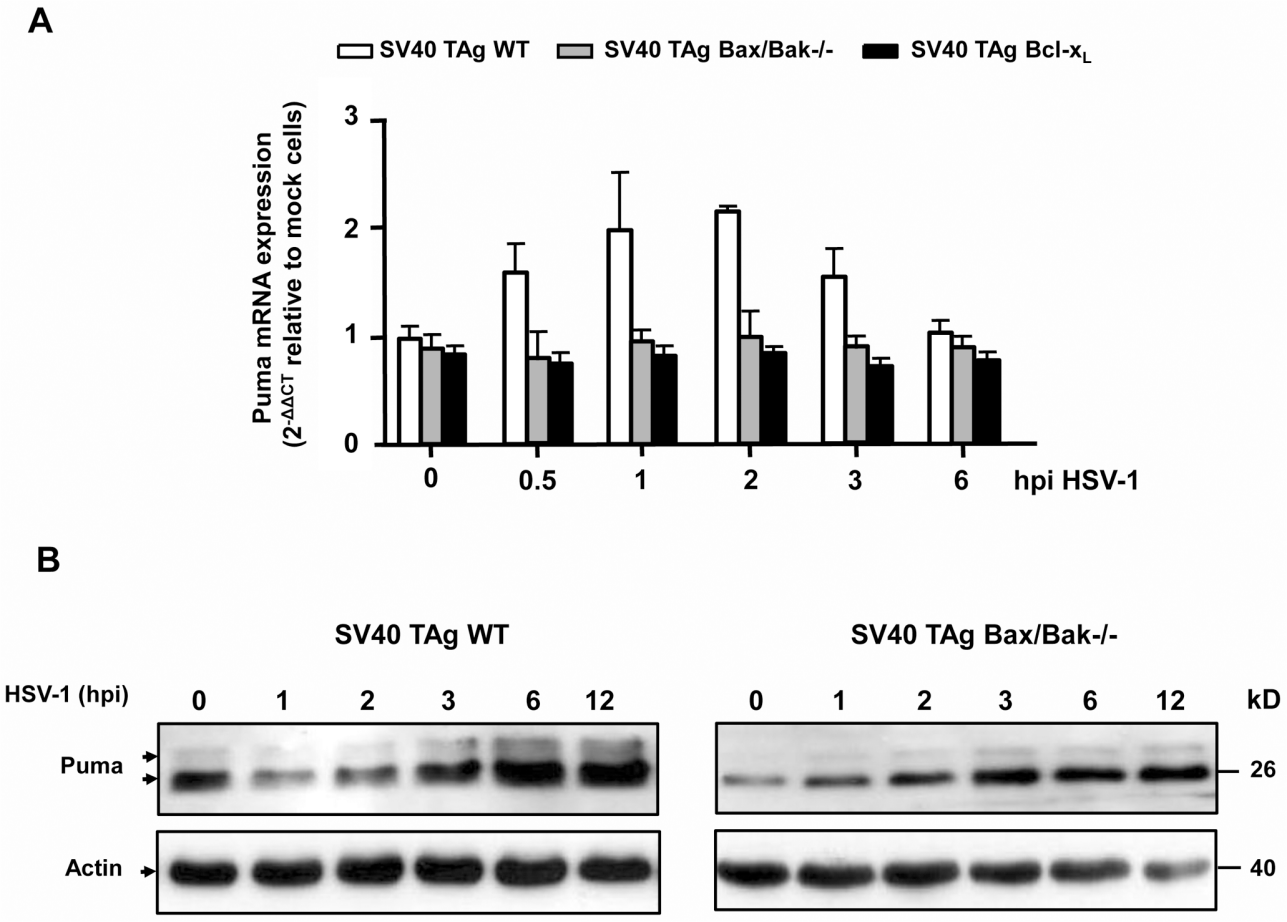
HSV-1 enhances both mRNA and protein levels of Puma, but mRNA increase occurs downstream of Bax/Bak-mediated MOMP. **(A)** Quantitative/real time reverse transcriptase PCR (qRT-PCR) of Puma mRNA isolated from SV40 TAg WT, Bax/Bak-/- and Bcl-x_L_ overexpressing MEFs infected with 10 moi of HSV-1 for 0, 0.5, 1, 2, 3 or 6 h. The mRNA values were normalized to the ribosomal housekeeping 18S gene and depicted as 2^-∆∆Ct^ relative to mock cells (see Materials and Methods for details). Data are the means of at least three independent experiments using three different clones of WT, Bax/Bak-/- and Bcl-x_L_ overexpressing cells ± SEM. The *p* values are the following: HSV-1 versus untreated: *p* = 0.05 for 0.5, 1 and 3 h, *p* = 0.01 for 2 h, n = 4. **(B)** Anti-Puma western blot analysis of total cell extracts of SV40 TAg WT and Bax/Bak-/- MEFs infected with HSV-1 for 0, 1, 2, 3, 6 or 12 h (hpi). Anti-actin as loading control.

We next wanted to know how HSV-1 impinges on Puma to activate Bax/Bak-mediated MOMP and apoptosis. Puma is known to be transcriptionally induced by p53, p73, Foxo3A, p65 NFκB and other transcription factors [41,42]. However, Puma can also be regulated at the posttranscriptional level, for example by phosphorylation at S10 [43,44]. We therefore first examined if Puma was transcriptionally induced by HSV-1 by performing a real time/quantitative reverse transcriptase PCR (qRT-PCR). As shown in Fig 8A, Puma mRNA levels were slightly increased after 2 h postinfection of SV40 TAg MEFs and decreased thereafter. However, surprisingly no increase of Puma mRNA was noted in MEFs deficient of Bax/Bak (SV40 TAg Bax/Bak-/- MEFs) or overexpressing Bcl-x_L_ (SV40 TAg Bcl-x_L_ MEFs) (Fig 8A, gray and black bars) indicating that changes in Puma transcription occurred after the initiation of HSV-1-induced apoptosis (MOMP). The same effect on Puma mRNA regulation was seen in HSV-1-infected WT and Bax/Bak-/- 3T9 MEFs (S5 Fig). Moreover, none of the classical Puma transcription factors p53, p73 or p65 NFκB were involved in HSV-1-induced apoptosis. As shown in S6 Fig, genetic ablation of p53 in 3T9 MEFs or p73 or p65 NFκB in SV40 TAg MEFs did not change the apoptosis rates after HSV-1 infection as compared to their respective WT cells. By contrast, Puma protein levels steadily increased after 3 h of HSV-1 infection of SV40 TAg WT cells, and this increase was independent of Bax/Bak (Fig 8B).

### Puma protein levels are also induced and required for SFV-induced, Bax/Bak-dependent cell death

**Fig 9:**
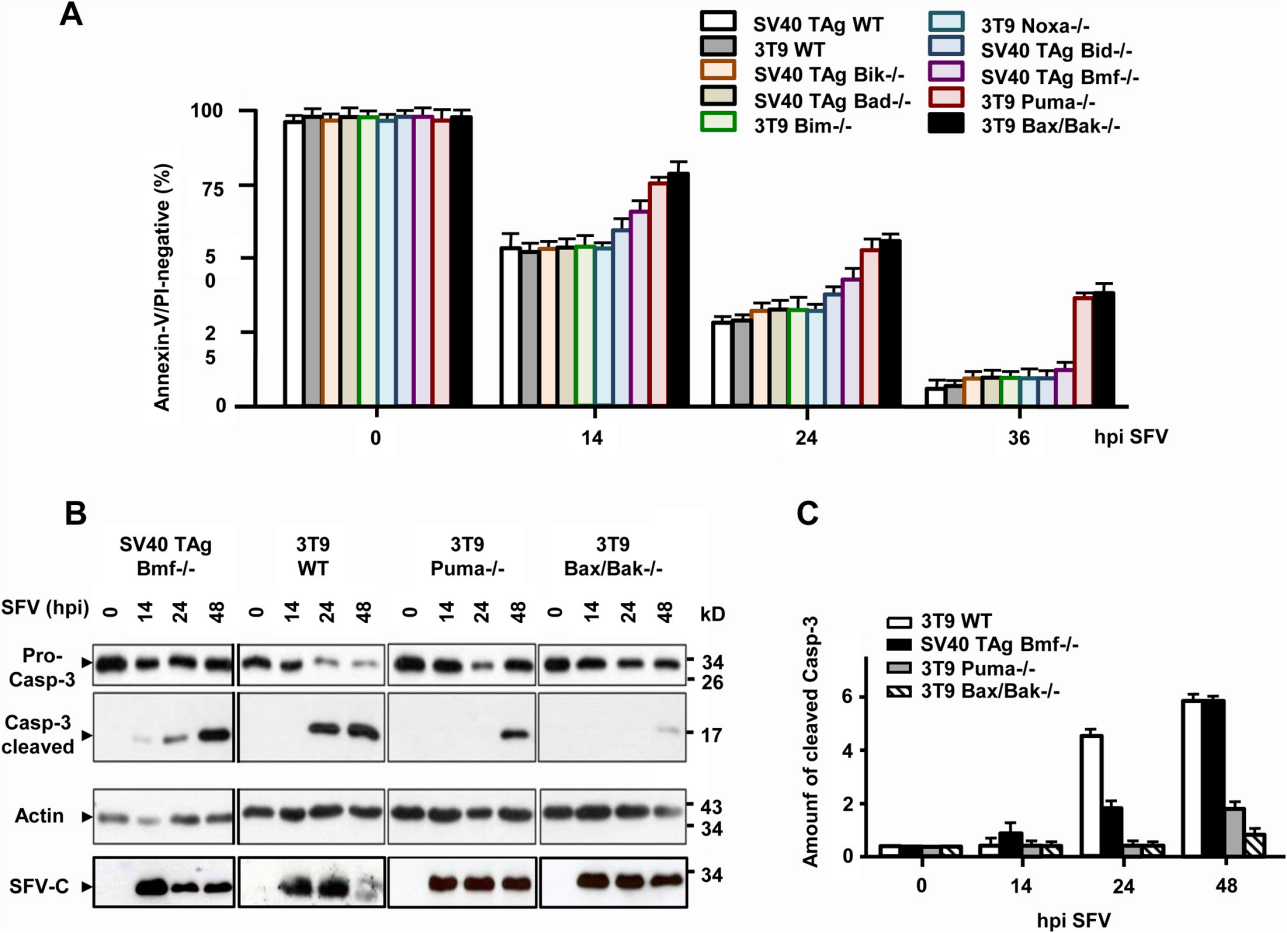
SFV-induced caspase-3 activation/processing and apoptosis require Puma and to a lesser extent Bmf. **(A)** Annexin-V/PI FACS and **(B/C)** anti-caspase-3 (pro-caspase-3 and cleaved caspase-3) western blot analyses of the various SV40 TAg-transformed or 3T9-immortalized WT and knock-out MEF cell lines/extracts infected with 10 moi of SFV for 0, 14, 24, 36 or 48 h (hpi). Anti-actin as loading and anti-SFV-C as infection controls in (B). The anti-cleaved caspase-3 bands in (B) are quantified by densitometric scanning, and the data are depicted in (C). Data in (A) and (C) are the means of at least three independent experiments using two clones of WT and each knock-out cell line ± SEM. The *p* values are the following: (A) SV40 TAg Bid-/- versus SV40 TAg WT: not significant; SV40 TAg Bmf-/- versus SV40 TAg WT: *p* = 0.05 for 14 and 24 h, not significant for 36 h; 3T9 Puma-/- versus 3T9 WT and 3T9 Bax/Bak-/- versus 3T9 WT: *p* < 0.001 for 14, 24 and 36 h, n = 4. (C) 3T9 Puma-/- and 3T9 Bax/Bak-/- versus 3T9 WT: *p* < 0.001 for 14 and 24 h; SV40 TAg Bmf-/- versus WT: *p* = 0.01 for 24 h, not significant for 48 h, n = 6.

**Fig 10:**
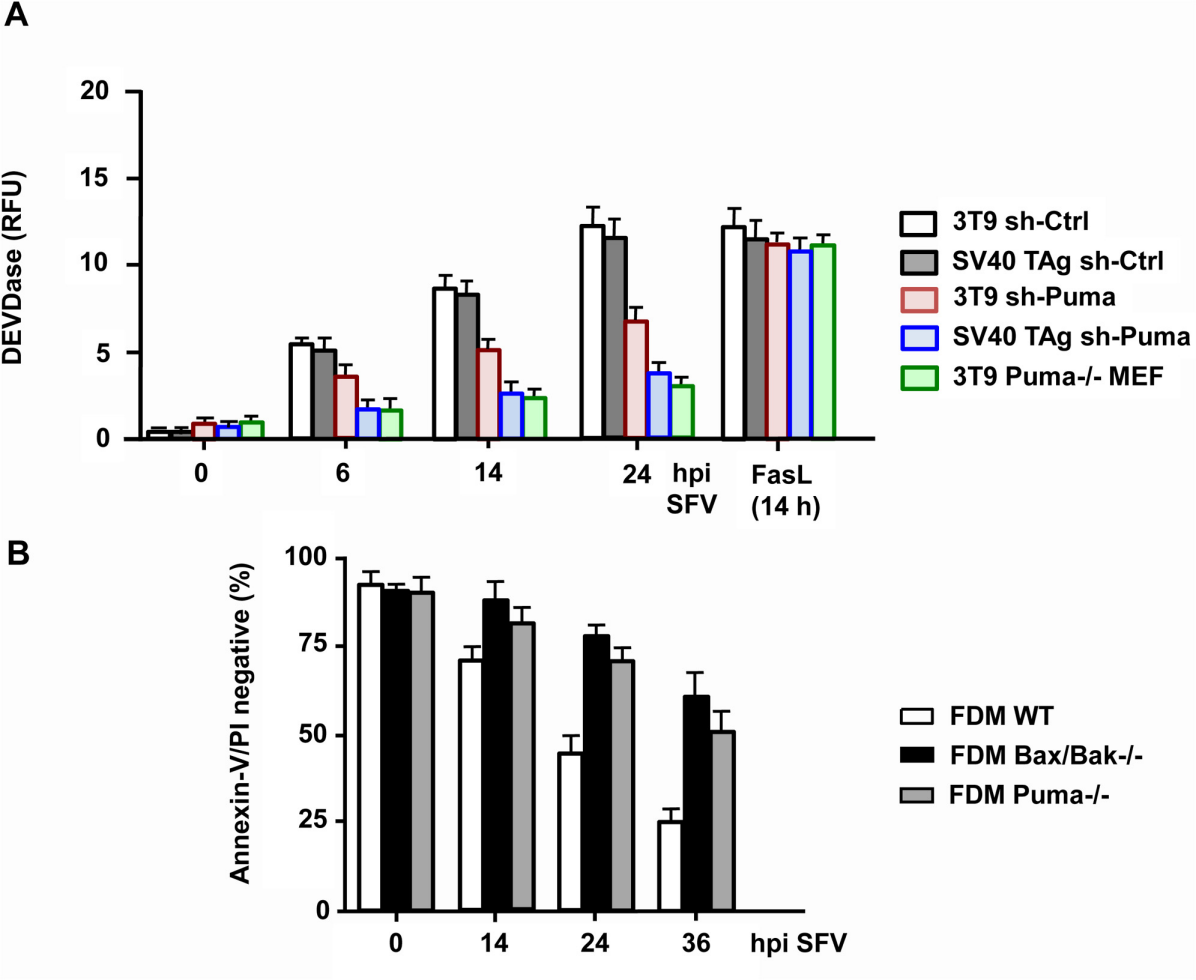
Puma knock-down in MEFs and its knock-out in FDMs also markedly diminish SFV-induced caspase-3 activation and apoptosis. **(A)** Caspase-3/-7 (DEVDase) activity assay of total extracts of puromycin selected, mixed populations of SV40 TAg-transformed and 3T9-immortalized MEFs infected with lentiviruses carrying either a scrambled shRNA (sh-Ctrl) or a shRNA of mouse Puma (sh-Puma), infected with SFV for 0, 6, 14 or 24 h (hpi). As a control the data of 3T9 Puma-/- MEFs are shown. In addition, the caspase activities are compared to those from cells treated with 10 ng/ml FasL for 14 h. Data are the means of at least three independent experiments ± SEM. The *p* values are the following: 3T9 sh-Puma versus 3T9 sh-Ctrl: *p* = 0.008 for 6 h, *p* < 0.001 for 14 and 24 h; SV40 TAg sh-Puma versus SV40 TAg sh-Ctrl and 3T9 Puma-/- versus 3T9 WT: *p* < 0.001 for 6, 14 and 24 h, n = 4. **(B)** Annexin-V/PI FACS analysis of WT, Puma-/- and Bax/Bax-/- FDM cells infected with 10 moi of SFV for 0, 14, 24 or 36 h (hpi). Data are the means of at least three independent experiments using three different clones of WT, Puma-/- and Bax/Bak-/- cells ± SEM. The *p* values are the following: Puma-/- versus WT: *p* = 0.03 for 14 h, *p* < 0.001 for 24 and 36 h. Bax/Bak-/- versus WT: *p* = 0.01 for 14 h, *p* < 0.001 for 24 and 36 h, n = 4.

We finally wanted to know if the requirement of Puma was unique to apoptosis induction by HSV-1 or if other unrelated viruses used the same BH3-only protein to kill their target cells. We recently reported that the positive sense, single stranded RNA virus Semliki Forest (SFV) induced Bax/Bak-dependent and-independent apoptosis of mammalian cells [32,33]. The BH3-only protein, which is responsible for Bax/Bak activation has not yet been unravelled in this infection system. We therefore used the same BH3-only deficient MEF cells for apoptosis analysis in response to SFV. As with HSV-1, MEF cells deficient for Bim, Bik, Bad and Noxa died in a similar way as WT cells after infection with 10 moi SFV for up to 48 h (Fig 9A). SV40 TAg Bmf-/- MEFs were slightly protected as were SV40 TAg MEFs devoid of Bid, as previously reported [32] (Fig 9A–9C). The best protection from SFV-induced apoptosis was however again observed in 3T9 Puma-/- MEFs. These cells resisted SFV-induced caspase-3 activation/processing (Figs 9B and 9C and 10A) and apoptosis (Fig 9A) to a similar extent as Bax/Bak-/- cells indicating that also SFV majorly used Puma to induce apoptosis via the intrinsic mitochondrial pathway. This finding was confirmed with 3T9 and SV40 TAg MEFs variants in which Puma expression was downregulated (although not entirely ablated) by shRNA (S4 Fig). Both Puma knock-down cells exhibited delayed caspase-3 activation kinetics in response to SFV as compared to their scrambled shRNA control cell lines (Fig 10A). Moreover, as with HSV-1, we compared the sensitivity of WT, Bax/Bak-/- and Puma-/- FDMs for SFV-induced apoptosis. Also for this cell type, the lack of Puma or Bax and Bak conferred a similar protection from SFV-induced apoptosis (Fig 10B).

**Fig 11:**
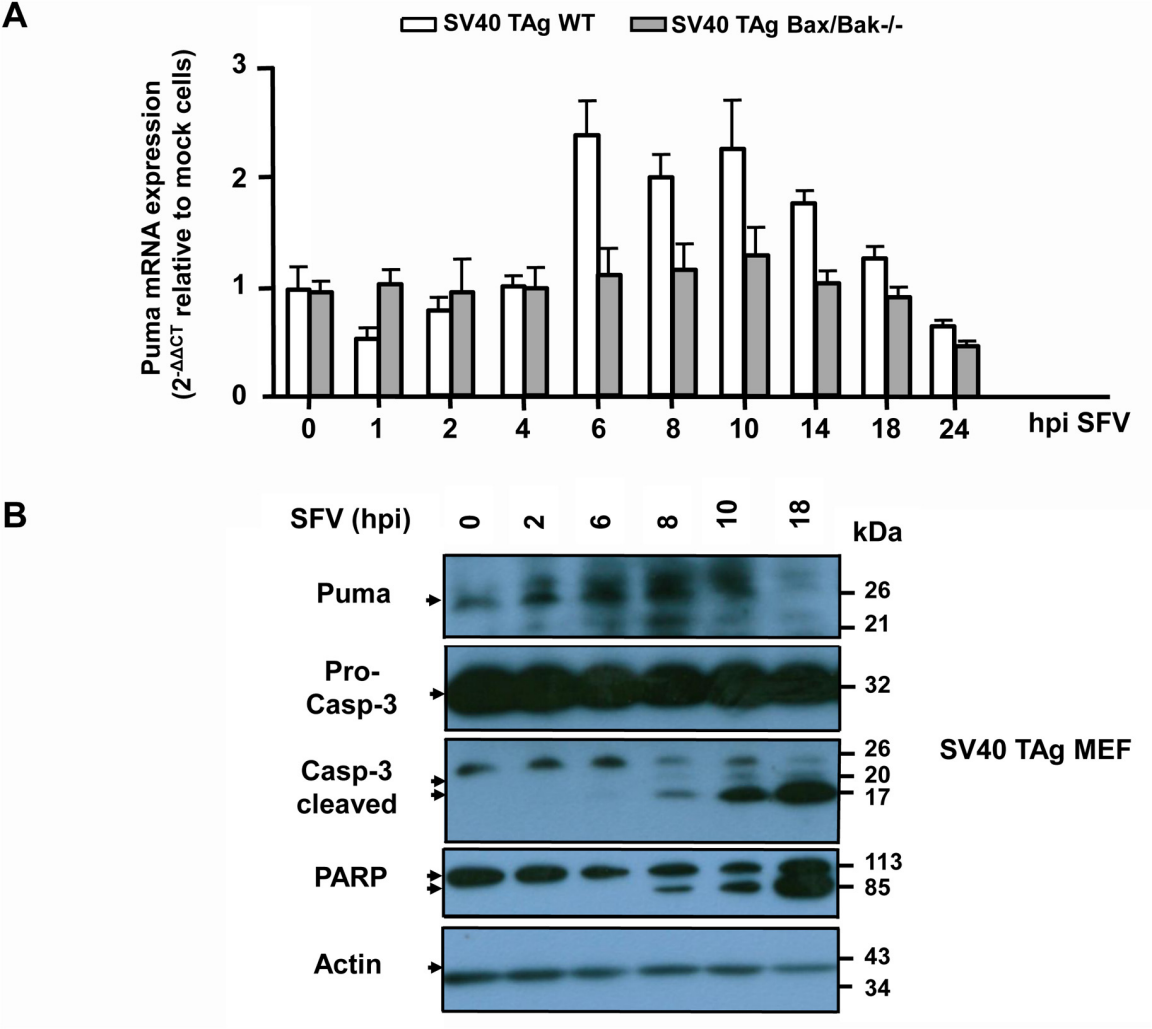
SFV enhances Puma mRNA and protein levels in MEFs and FDM cells, but mRNA increase is late and Bax/Bak-dependent. **(A)** Quantitative/real time reverse transcriptase PCR (qRT-PCR) of Puma mRNA isolated from SV40 TAg WT and Bax/Bak-/- MEFs infected with 10 moi of SFV for 0, 1, 2, 4, 6, 8, 10, 14, 18 or 24 h (hpi). The mRNA values were normalized to the ribosomal housekeeping 18S gene and depicted as 2^-∆∆Ct^ relative to mock cells (see Materials and Methods for details). Data are the means of at least three independent experiments ± SEM. The *p* values are the following: SFV-treated WT versus untreated: *p* = 0.005 for 6 h, *p* = 0.01 for 8 h, *p* = 0.008 for 10 h. SFV-treated Bax/Bak-/- versus untreated: not significant, n = 5. **(B)** Anti-Puma, anti-caspase-3 (pro-caspase-3 and cleaved caspase-3) and anti-PARP western blot analysis of total cell extracts of SV40 TAg WT MEFs infected with SFV for 0, 2, 6, 8, 10 or 18 h. Anti-actin as loading control.

Considering the crucial role of Puma for SFV-induced apoptosis, we wanted to know if Puma is similarly regulated as in HSV-1 infections. We first performed a qRT-PCR of Puma from WT and Bax/Bak-/- SV40 TAg MEFs infected with 10 moi of SFV for up to 24 h. As shown in Fig 11A, Puma mRNA increased in both cell lines after 6 h postinfection but again this response was entirely dependent on Bax/Bak. By contrast, as with HSV-1, the amount of Puma protein started to increase after 2 h of SFV infection of SV40 TAg MEFs, and it stayed high until 10 h after which it was degraded, probably by a caspase-dependent process (Fig 11B). Importantly, Puma protein stabilization occurred before caspase-3 processing and PARP cleavage, which was only detected after 8 h postinfection (Fig 11B) reinforcing the notion that Puma was a key mediator of SFV-induced Bax/Bak activation, MOMP and apoptosis.

## Discussion

While mechanisms utilized by viruses to prevent premature apoptotic death of their host cells are well known, relatively few data are available on molecular events that initiate and drive an apoptotic innate response to virus infection. In this study we show for the first time that two phylogenetically distant viruses, such as the double stranded DNA virus HSV-1 and the single stranded RNA virus SFV, use the same BH3-only protein, Puma, to trigger Bax/Bak-mediated MOMP, effector caspase-3/-7 activation and apoptosis of different mammalian cell types. In both scenarios, Puma transcription is increased, but as a consequence rather than a cause of Bax/Bak-induced MOMP. Moreover, p53, p73 and p65 NFκB, the major transcriptional inducers of Puma expression, are not involved in HSV-1-induced apoptosis. Instead Puma protein levels increased rapidly after infection of both viruses in a Bax/Bak-independent fashion suggesting that a yet unknown molecular mechanism to stabilize the Puma protein is crucial for virus-induced apoptosis.

We firmly believe that our finding uncovers a more general mechanism by which viruses induce apoptosis of their host cells. Cruz et al. [38] reported that the measles virus V protein inhibits p73 and decreases the abundance of Puma. Moreover, very recently, Puma protein levels were shown to be increased after infection with influenza A virus [45]. However none of the previous studies used Puma knock-out or knock-down systems to prove the involvement of Puma in virus-induced apoptosis. Here we show that three different cell lines, mouse embryo fibroblasts, factor-dependent monocytes as well as human colon carcinoma cells all require Puma to succumb to HSV-1- and SFV-induced cell death. This is not only demonstrated with established Puma-/- cells, which may have acquired mutations in other genes leading to apoptosis resistance, but also in freshly prepared cells in which Puma was downregulated by shRNA. Moreover, the fact that Puma-/- cells are as resistant to virus-induced cell death as Bax/Bak-/- cells indicates that Puma is the major BH3-only protein mediating Bax/Bak activation in response to HSV-1 and SFV infections. The only other BH3-only protein, which seems to partially contribute to this process is Bmf since Bmf-/- cells displayed a slight protection to apoptosis by both viruses during early phases of infection (24 h). Munger and Roizman previously suggested the BH3-only protein Bad as a mediator of HSV-1-induced apoptosis due to the fact that the US3 protein kinase of HSV-1 could phosphorylate and inactivate Bad and protect cells from Bad-induced cell death [15]. However, Bad-/- cells were not studied in their report and as shown here Bad-/- and WT MEFs died in a similar way after HSV-1 infection. Another controversial issue has been to what extent death receptor signalling contributes to HSV-1- and SFV-induced apoptosis. While in one case inhibition of FasL by soluble Fas did not prevent apoptosis caused by HSV [46], another report found that HSV-induced apoptosis was suppressed by antibodies directed towards Fas or FasL [47,48]. Moreover, gD and gJ of HSV-1 were shown to protect against FasL-induced apoptosis [17,18] and dendritic cells seem to die after HSV-1 infection due to the downregulation of the caspase-8 inhibitor c-FLIP [28]. However, all these findings provided only indirect evidence for or against a role of FasL/Fas signalling in HSV-1-induced apoptosis and the other death receptor signalling systems have not been studied. Here we used neutralizing antibodies or recombinant Fc proteins to clearly show that neither FasL, TNFα, TRAIL nor their receptors were required for the death of HSV-1-infected cells. We previously published that this was also not the case for SFV [32]. Finally, we used the RIP1 inhibitor necrostatin-1 and shRNA-mediated downregulation of RIP3 to exclude the participation of necroptosis in HSV-1-induced cell death. We can therefore not confirm a recent report by Wang et al. [49] that HSV-1 triggers necrosis/necroptosis via RIP3, at least not in our cellular systems.

Our study therefore shows that Puma is the major sentinel/sensor of incoming viruses to convey an apoptotic signal to MOMP. But how does Puma sense viral infection and/or what is the viral component, if any, which engages Puma? One possibility is via the transcriptional regulation of Puma. We previously reported that the gD envelope protein of HSV-1 induces the transcription factor NFκB [18,19,23]. A variety of other viruses elicit the stabilization and activation of p53 or p73 [6] and SFV was found to provoke ER stress and the subsequent activation of the transcription factor CHOP/CBP through the overproduction of envelope proteins in the ER lumen [50]. All these transcription factors are known to induce Puma expression [41,42]. We indeed measured increased Puma mRNA levels after both HSV-1 and SFV infections. However, surprisingly, the increase in Puma mRNA levels was dependent on Bax/Bak because it was not detected in MEFs deficient of Bax/Bak or overexpressing Bcl-x_L_. This indicates that Puma transcription in response to HSV-1 and SFV infection is not an early event of apoptosis but occurs later under the control of MOMP and caspase-3/-7 activation. Perhaps a substrate that is cleaved by caspase-3/-7 directly or indirectly triggers Puma transcription therefore stimulating a feed-forward loop to amplify virus-induced apoptosis. Consistent with this notion, the genetic deletion of p53, p73 or p65 NFκB in MEFs did not protect the cells from HSV-1 or SFV-induced apoptosis although cell death was slightly delayed in both cases (S6 Fig and data not shown). Moreover, in both SV40 TAg-transformed and 3T9-immortalized MEFs as well as in U937 cells, the p53 function is compromised so that cell death measured in these cells cannot be p53-mediated. Finally, we previously reported that SFV-induced apoptosis does not proceed via an ER stress response because SFV replicons, which do not produce envelope proteins in infected cells, trigger apoptosis as efficiently as native viruses [32].

Our data rather indicate that a posttranslational regulation of the Puma protein is responsible for conveying the viral death signal to Bax/Bak. Puma is already expressed on the endogenous level in healthy MEFs, FDMs and a variety of other cells. To prevent accidental Bax/Bak activation in healthy cells, the pro-apoptotic activity of Puma must be inhibited. On one hand it is known that Puma is sequestered by Bcl-2-like survival factors [35]. On the other hand Puma was shown to be rapidly degraded after phosphorylation at several serine residues [43,44,51]. In particular Ser10 was phosphorylated by the IKK1/IKK2/Nemo complex in response to growth factor/cytokine stimulation leading to the ubiquitination and proteasomal degradation of the Puma protein [44]. Since IKK is an upstream kinase crucial for NFκB activation [52], HSV-1 may use this mechanism to keep Puma levels low in certain cells such as U937 monocytes (for example via gD). Furthermore, Carpenter et al. [51] recently reported on the phosphorylation of Puma on three tyrosine residues by the HER2 receptor tyrosine protein kinase, which also destabilized the Puma protein. We have not yet studied the phosphorylation status or any other posttranslational modification of Puma in uninfected and HSV-1- or SFV-infected cells. Experiments are underway to determine if such a modification of Puma increases its protein stability and pro-apoptotic activity in infected cells.

SFV does not encode for any death protective proteins. This explains why the virus is a potent inducer of apoptosis in a variety of mammalian cell types and is currently used as a vector for anti-cancer therapy. The benefit of eliminating infected cells would be to prevent their presentation to the immune system. Since the virus reproduces and forms progeny before killing the cells [32], it could further propagate without being fully neutralized. The question remains if it is a viral component such as dsRNA or any of the non-structural proteins nsp1-4, which trigger a signalling cascade leading to Puma protein stabilization or if infected host cells start to produce or activate cellular factors which then impinge on Puma. By contrast, HSV-1 expresses a variety of potential cell survival factors and activates numerous cellular proteins such as for example NFκB, which can inhibit apoptosis. Most cells that are infected by HSV-1 are therefore not necessarily killed but survive to sustain viral reproduction. This is the case for human U937 monocytes. Indeed, when NFκB is inhibited in these cells, they become more sensitive to HSV-1-induced apoptosis. Although we have not yet examined the role of Puma in these cells, we could clearly show that HSV-1-induced apoptosis is Bax/Bak-mediated. This is the first formal proof that HSV-1 triggers apoptosis in human cells via Bax/Bak-mediated MOMP and explains our previous report that overexpression of Bcl-2 could protect U937 cells from HSV-1-induced apoptosis [24]. Obviously in mouse cells (MEFs and FDMs) the NFκB pathway is not as active as in human monocytes, so that these cells could serve as an ideal model system to dissect the apoptotic signalling induced by HSV-1. As with SFV, we need now to understand which viral component of HSV-1 or which cellular factor induced by the virus leads to Puma protein stabilization and Bax/Bak-mediated MOMP and apoptosis.

## Materials and Methods

### Antibodies and Reagents

Rabbit polyclonal anti-caspase-3 antibodies recognizing the 32 kD proform (#9661) and the cleaved active 17 kD form (#9662) were purchased from Cell Signaling, rabbit polyclonal anti-Puma from ProSci, mouse monoclonal anti-actin (clone C4) from BD Biosciences, mouse monoclonal anti-COX-VIc from Life Technologies, mouse monoclonal anti-cytochrome c (clone 6H2.B4), horseradish peroxidase-conjugated anti-rabbit or anti-mouse secondary antibodies from Jackson Immunoresearch Laboratories, rabbit polyclonal anti-Bax (NT), rabbit polyclonal anti-Bak (06–536), neutralizing anti-human CD95/Fas antibody (clone ZB4) and FITC-conjugated goat anti-mouse IgG from Millipore and PE-conjugated goat anti-rabbit IgG, F(ab’)_2_ fragments from Santa Cruz Biotechnology. Rabbit polyclonal anti-RIP3 (R4277), Hoechst 33334, propidium iodide (PI), actinomycin D (Act D), bovine serum albumin (BSA), Triton X-100 (TX) and NP40 were bought from Sigma-Aldrich, the caspase inhibitors Q-VD-OPH and ZVAD-fmk from Biomedicals, lipofectamine from Invitrogen and the fluorogenic caspase-3 substrate DEVD-AMC (acetyl-Asp(OMe)-Glu(OMe)-Val-Asp(OMe)-7-amino-4-methylcoumarin) from Alexis Biochemicals. Acrylamide and dithiothreitol (DTT) were from AppliChem, Necrostatin-1 (Nec-1) from Calbiochem and PageRuler Prestained Protein Ladder from Thermo Scientific. Recombinant mouse TNFα and neutralizing monoclonal anti-mouse TNF-R1 antibodies were purchased from R&D Systems. Lentiviral shRNAs to knock-down mouse Puma, mouse RIP3 and the respective scrambled shRNAs (sh-Ctrl) were obtained from Sigma-Aldrich. Recombinant CD95/FasL was kindly provided by P. Schneider, Lausanne, Switzerland, mouse CD95/Fas-Fc by T. Brunner, Konstanz, Germany, and leucine zipper-tagged TRAIL (lz-TRAIL), TRAIL-R2-Fc and the F(ab)’2 fragment of a neutralizing anti-TRAIL-R1 antibody by H. Walczak, London College, UK). The monoclonal anti-gD antibody was kindly provided by G. Cohen and R. Eisenberg, University of Pennsylvania, Philadelphia, PA, the polyclonal anti-SFV-capsid (C) antibody by J. Pavlovic, Zürich, Switzerland.

### Cells

SV40 large T antigen (TAg)-transformed WT, Bid-/- [53], Bad-/- [54], and Bax/Bak-/- mouse embryo fibroblasts (MEFs) [55] were obtained from the late Korsmeyer lab, Dana Farber Cancer Institute, Boston, MA, USA. WT and SV40 TAg-transformed Bmf-/- MEFs were from Andreas Villunger, Innsbruck, Austria [56]. WT and SV40TAg-transformed Bik-/- [57] and p65 NFκB-/- MEFs [58] as well as 3T9-immortalized WT, Bax/Bak-/- [59], Puma-/-, Noxa-/-, Bim-/-, p53+/- and p53-/- MEFs [60] were kindly provided by Andreas Strasser, WEHI, Melbourne, Australia. WT, Puma-/- and Bax/Bak-/- mouse Factor (IL-3)-Dependent Monocytes (FDMs) were a generous gift from Paul Ekert, WEHI, Australia [61], and the SV40 TAg-transformed WT and p73-/- MEFs came from Gerry Melino, Rome, Italy [62]. Human colon carcinoma HCT116 WT, Bax/Bak-/- and Puma-/- cells were originally generated in Bert Vogelstein’s lab, Howard Hughes Medical Institute, Baltimore, MD, USA and provided by Peter Daniel, Charité Berlin, Germany [63–65]. SV40 TAg-transformed MEFs overexpressing Bcl-x_L_ were generated as described [33]. Jurkat cells were bought from ATCC. Mixed populations of stable U937 pcDNA3 and U937 mIκBα human monocytes were generated by transfecting them with either the pcDNA3 vector alone (Life Technologies) or with pDNA3 containing the non-phosphorylatable mIκBα mutant [18]. U937 stably expressing the pMEP vector (U937 pMEP) or mouse Bcl-2 (U937 Bcl-2) were generated in Borner et al., 1996 [66]. The Puma or RIP3 knock-down and respective control cells were created by infecting SV40 TAg-transformed and/or 3T9-immortalized MEFs with lentiviruses containing either scrambled, mouse Puma or mouse Rip3 shRNAs, as described in [33]. Similarly, U937 control and Bax/Bak single and double knock-downs were generated by lentiviral infections with respective human Bax, Bak or scrambled shRNAs [67]. All the cells were grown in high-glucose Dulbecco’s modified Eagle’s medium (DMEM) (4.5 g/l glucose) (PAA) supplemented with 10% fetal bovine serum (FBS) (Lonza). Vero African green monkey kidney cells (ATCC) were propagated in MEM (HyClone Europe, Cramlington, UK) containing 6% FBS (Lonza) at 37°C in a CO_2_ incubator. The insect cell line Aedes albopictus was maintained at 28°C in L15 medium (Gibco) supplemented with 10% fetal calf serum (FCS) and 4% Difco Bacto phosphate tryptose broth.

### Virus production and titer determination

The F-strain of HSV-1 (originally obtained from ATCC) and the virulent L10 strain of SFV [33] were used throughout this study. Virus stocks were either produced in Vero cells (HSV-1) or in mosquito *A*. *albopictus* cells (SFV) [33]. Virus titers were determined by the plaque assay as previously described [33]. Both viruses were stored in aliquots at -80°C in titers of 1–2 x 10^9^ PFU/ml.

### Cell death assays

SV40 TAg-transformed or 3T9-immortalized MEFs, mouse monocytic FDMs, human monocytic U937 cells and human colon carcinoma HCT116 cells were infected with 10 moi of SFV or 50 moi of HSV-1 while shaking in DMEM plus 0.5% FCS. After 1 h at 37°C, viral infection was stopped and the cells incubated in DMEM plus 10% FCS until processed for further experiments. Alternatively, the cells were treated with 50 ng/ml FasL (kindly obtained from Pascal Schneider, Lausanne), 10 ng/ml TNFα (R&D Systems) or 500 ng/ml leucine zipper (lz) TRAIL (kindly provided by Henning Walczak, London). Apoptosis was quantified by annexin-V-GFP/propidium iodide (PI) FACS analysis using a FACS Calibur equipment from Becton Dickinson, and caspase-3/-7 activity was measured by the DEVDase assay as described [68]. Fluorescence was detected in the Fluoroskan Ascent equipment (Thermo Labsystems) and the relative fluorescence units (RFUs) were normalized to the protein concentration.

### Neutralization of death ligands and/or their receptors

To study the implication of CD95/Fas, TRAIL-R1, TNF-R1 or their respective ligands CD95/FasL, TRAIL or TNFα, we incubated human Jurkat cells with neutralizing antibodies against human Fas (anti-hCD95/Fas) or MEFs with neutralizing antibodies against mouse TRAIL-R1 (anti-mTRAIL-R1 Fab2) or mouse TNF-R1 (anti-mTNF-R1) or recombinant receptor Fc proteins directed against mouse FasL (mCD95/Fas-Fc) or mouse TRAIL (mTRAIL-R2-Fc) at 37°C for 1 h before either adding the respective ligands or infecting the cells with 10 moi of HSV-1 for up to 24 h, exactly as described for SFV [32].

### Protein extraction and western blot analysis

For total extracts, cells were collected on ice by scraping. The cells were washed with ice-cold 1 x PBS and lysed in buffer A (25 mM HEPES KOH, pH 7.4, 2 mM MgCl_2_, 2 mM EGTA) containing 1% of Triton-X-100 or in buffer B (50 mM TrisHCl, pH 8, 150 mM NaCl) containing 1% NP40 and complete protease inhibitors (Roche). After 20 min incubation on ice, the suspension was centrifuged at 4°C and 13000 rpm for 5 min. The supernatant contained the cytosolic and TX- or NP40-solubilized membrane proteins. Protein concentration was determined using Bradford assay. An amount of 80 μg of proteins was boiled in SDS sample buffer (50 mM Tris-HCl, pH 6.8, 10 mM dithiothreitol, 2% SDS, 0.1% bromophenol blue, 10% glycerol), and then subjected to electrophoresis on 12.5–15% denaturing polyacrylamide gel followed by transferring to nitrocellulose membrane. The membranes were blocked in 1 x PBS supplemented with 5% non-fat dried milk and 0.05% Tween-20 for 1 h and incubated with primary antibodies at 4°C overnight. After three washings in PBS, secondary horseradish peroxidase-conjugated antibodies were added and the membranes incubated at RT for 90 min. Proteins were visualized with the enhanced chemiluminescence SuperSignal West Pico Chemiluminescent.

### Immunofluorescence

5 x 10^3^ MEFs were grown on glass coverslips in multiwell plates and infected with 10 moi of HSV-1 or SFV. At 18h post-infection, the cells were fixed in 4% paraformaldehyde and permeabilized in 0.1% Triton X-100 (Sigma). The cells were then incubated with mouse monoclonal anti-gD or mouse monoclonal anti-cytochrome c and rabbit polyclonal anti-caspase-3 antibodies followed by FITC-conjugated goat anti-mouse IgG, (Millipore) and PE-conjugated goat anti-rabbit IgG, F(ab’)_2_ fragment (Santa Cruz Biotechnology) secondary antibodies (1:200) for 90 min. Nuclei were stained with Hoechst 33334 (5 mg/ml; Sigma). The samples were directly viewed under a Leica DMRE fluorescence microscope.

### RNA Extraction, Reverse Transcription, and Quantitative Polymerase Chain Reaction (PCR)

Total RNA was extracted from 1 x 10^6^ mock- or SFV- or HSV-1-infected WT or knock-out MEFs using the Trizol reagent (Invitrogen) according to the manufacturer’s instructions. The amount of total RNA isolation was quantified by optical density at 260 nm. 1–2 μg of total RNA was reverse transcribed into cDNA using the High-Capacity cDNA Transcription kit (Applied Biosystems, Carlsbad, CA, USA) according to the manufacturer’s instructions. To digest the leftover RNA, 1 μl RNase H was added and incubated at 37°C for 20 min. Quantitative polymerase chain reaction (qPCR) amplification of the various cDNAs was carried out in a 25 μl solution containing 11.25 μl SYBR Green PCR Master Mix (Bio-Rad laboratories), 150 nM forward and reverse primers, 750 ng cDNA template, 10.25 μl H_2_O and the Hotstar meteor Taq polymerase. The sequences of the primer used for the qPCR were: Puma forward murine primer 5’-GCCCAGCAGCACTTAGAGTC- 3'; Puma reverse murine primer 5'- GGTGTCGATGCTGCTCTTCT -3’; 18S forward primer 5’-GTAACCCGTTGAACCCCATT-3’; 18S reverse primer 5’CCATCCAATCGGTAGTAGCG-3’. Samples were heated for 10 min at 95°C and then subjected to 45 cycles of PCR amplification, each cycle consisting of 15 s at 95°C and 60 s at 60°C. Within each experiment, a no-template control and the housekeeping 18S gene were run in parallel. Each run was completed with a melting curve analysis to confirm the specificity of amplification and lack of unspecific products and primer dimers. Quantification was performed using the Ct comparative method. The relative gene mRNA levels were calculated as follows: ΔCt values (Ct (gene of interest)—Ct (internal control housekeeping gene 18S)) represent the difference, in threshold cycle number, between genes of interest and the housekeeping gene 18S. To compare the expression of different genes, ΔCt values were normalized to the mean value of ΔCt from the least expressed gene (2^-ΔΔCt^ = 2^-(ΔCt (gene of interest)—mean value of the less expressed gene ΔCt (normalizer))^).

### Statistics

Statistical significance (*p* values) was analyzed by a two-tailed Student’s t test. Data are the means of at least three experiments using two to three independent cell clones ± SEM.

## Supporting Information

S1 Fig Original annexin-V/PI FACS dot plot data of HSV-1-infected U937 cells, SV40 TAg MEFs, FDM and HCT116 cells. A representative set of original annexin-V/PI FACS dot plot data of U937 cells carrying the pcDNA3 vector or expressing a dominant-negative version of IκBα (mIκBα) **(A)**, SV40 TAg WT or Bax/Bak-/- MEFs **(B)**, WT, Bax/Bak-/- or Puma-/- FDMs **(C)**, or WT, Bax/Bak-/- or Puma-/- HCT116 cells **(D)**, infected with 50 (A) or 10 (B-D) moi of HSV-1 in the presence or absence of 25 μM of the general caspase inhibitor QVD for 0 (mock), 12, 24, 36 or 48 h (hpi). The x-axis (FL1-H) shows annexin-V-GFP, the y-axis (FL2-H) shows PI staining. The lower left quadrant depicts the percentage of double negative, surviving cells, the lower right quadrant the percentage of single positive (annexin-V) apoptotic cells and the upper right quadrant the percentage of double positive (annexin-V/PI) secondary necrotic cells.
(TIF)

S2 Fig HSV-1 effectively infects SV40 TAg-transformed mouse embryo fibroblasts (MEFs) and produces high viral titers, especially when Bax/Bak are depleted. **(A)** Anti-env gD immunofluorescence analysis of SV40 TAg WT and Bax/Bak-/- MEFs infected with 10 moi of HSV-1 for 16 h (hpi). gD positivity represents viral infection, Hoechst 33334 stains nuclear DNA. **(B)** The number of gD positive cells in (A) were determined by counting 10 different fields under the fluorescent microscope. The data represent the means of 3 independent stainings (counting 10 fields each) ± SEM. The *p* values are the following: HSV-1 versus mock, *p* < 0.001 for 24 and 48 h; HSV-1-infected Bax/Bak-/- versus HSV-1-infected WT cells: *p* = 0.01 for 24 h, *p* = 0.05 for 48 h, *p* < 0.001 for 72 h, n = 5. **(C)** Viral titers determined by the plaque assay and depicted as Log_10_ Plaque Forming Units (PFU)/ml after infecting U937 vector control (pMEP) and Bcl-2-overexpressing (Bcl-2) monocytes with 50 moi or infecting SV40 TAg WT and Bax/Bak-/- MEFs with 10 moi of HSV-1 for up to 72 h. Data are the means of at least three independent experiments ± SEM. The *p* values are < 0.001 for U937 Bcl-2 versus pMEP and SV40 TAg Bax/Bak-/- versus WT at 48 and 72 hpi, n = 4.
(TIF)

S3 Fig Effective knock-down of RIP3 in SV40 TAg-transformed WT and Bax/Bak-/- MEFs by lentiviral transduction of shRNA. Anti-RIP3 western blot analysis of total extracts from mixed populations of puromycin-selected, SV40 TAg WT and Bax/Bak-/- MEFs infected with lentiviruses carrying a scrambled shRNA (sh-Ctrl) or an shRNAs for mouse RIP3 (sh-Rip3). Anti-actin as loading control.
(TIF)

S4 Fig Effective knock-down of Puma in SV40 TAg-transformed and 3T9-immortalized MEFs by lentiviral transduction of shRNA. Anti-Puma western blot analysis of total extracts from mixed populations of puromycin-selected, SV40 TAg-transformed and 3T9-immortalized MEFs infected with lentiviruses carrying a scrambled shRNA (sh-Ctrl) or shRNAs for mouse Puma (Sigma Open Labs). For comparison, an extract from 3T9 Puma-/- MEFs is shown. Anti-actin as loading control.
(TIF)

S5 Fig HSV-1 enhances Puma mRNA levels in 3T9 MEFs in a Bax/Bak-dependent manner. Quantitative/real time reverse transcriptase PCR (qRT-PCR) of Puma mRNA isolated from 3T9-immortalized WT and Bax/Bak-/- MEFs infected with 10 moi of HSV-1 for 0, 0.5, 1, 2, 3, 6, 12, 18 or 24 h. The mRNA values were normalized to the ribosomal housekeeping S18 gene and depicted as 2^-∆∆Ct^ relative to mock cells (see Materials and Methods for details). Data are the means of at least three independent experiments using three different clones of 3T9 WT and Bax/Bak-/- cells ± SEM. The *p* values are the following: HSV-1 versus untreated: *p* = 0.05 for 0.5 and 6 h, *p* = 0.01 for 1, 2 and 3 h, n = 3.
(TIF)

S6 Fig HSV-1-induced apoptosis does not require p53, p73 or p65 NFκB. Annexin-V/PI FACS analysis of **(A)** 3T9-immortalized WT, p53-/+ and p53-/- MEFs, **(B)** SV40 TAg-transformed WT and p73-/- MEFs or **(C)** SV40 TAg-transformed WT and p65 NFκB-/- MEFs, infected with 10 moi of HSV-1 for 0, 14, 24 or 36 h (hpi). In (A) and (B) the cells were also exposed to UV light (100 J/m^2^) for 24 h as a positive control. Data are the means of at least three independent experiments using two different clones of WT and knock-out cells ± SEM. The *p* values are < 0.001 for UV-treated p53-/- versus WT and UV-treated p73-/- versus WT MEFs, n = 3.
(TIF)
