# Supplementary figures and images for: Phylogenetically Distant Viruses Use the Same BH3-Only Protein Puma to Trigger Bax/Bak-Dependent Apoptosis of Infected Mouse and Human Cells
Source: PLoS One. 2015 Jun 1;10(6):e0126645. doi: 10.1371/journal.pone.0126645 (PMC4452691; doi:10.1371/journal.pone.0126645)

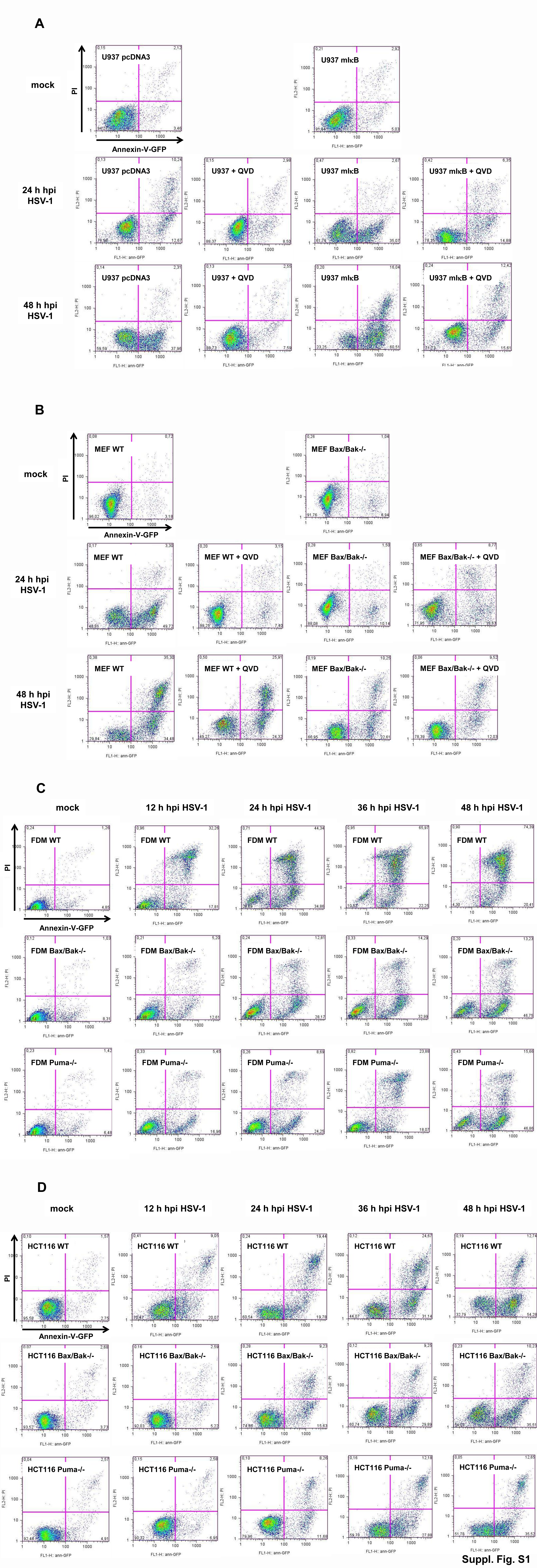

Supplement: S1 Fig — A representative set of original annexin-V/PI FACS dot plot data of U937 cells carrying the pcDNA3 vector or expressing a dominant-negative version of IκBα (mIκBα) (A), SV40 TAg WT or Bax/Bak-/- MEFs (B), WT, Bax/Bak-/- or Puma-/- FDMs (C), or WT, Bax/Bak-/- or Puma-/- HCT116 cells (D), infected with 50 (A) or 10 (B-D) moi of HSV-1 in the presence or absence of 25 μM of the general caspase inhibitor QVD for 0 (mock), 12, 24, 36 or 48 h (hpi). The x-axis (FL1-H) shows annexin-V-GFP, the y-axis (FL2-H) shows PI staining. The lower left quadrant depicts the percentage of double negative, surviving cells, the lower right quadrant the percentage of single positive (annexin-V) apoptotic cells and the upper right quadrant the percentage of double positive (annexin-V/PI) secondary necrotic cells. (TIF) [file pone.0126645.s001.tif]

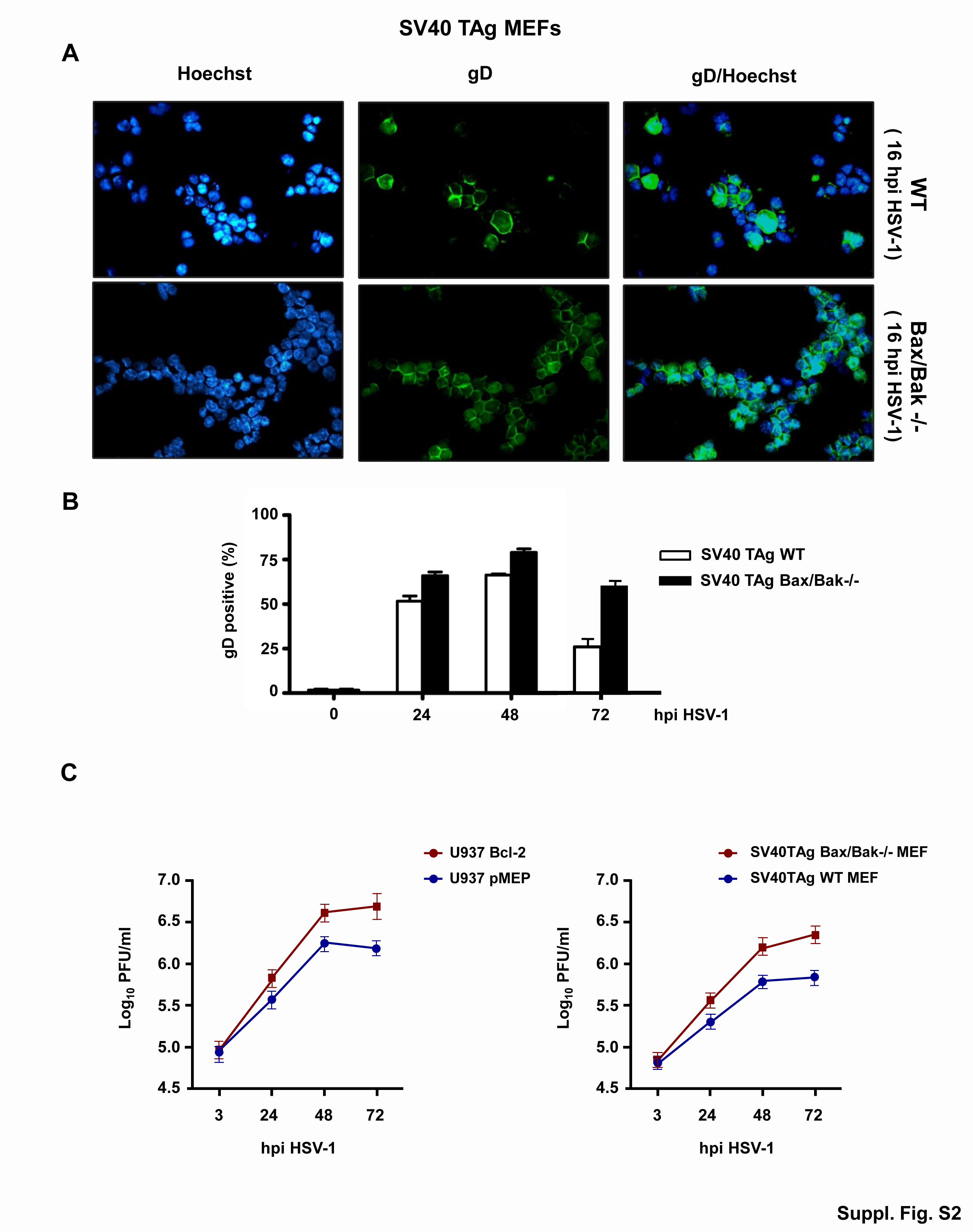

Supplement: S2 Fig — (A) Anti-env gD immunofluorescence analysis of SV40 TAg WT and Bax/Bak-/- MEFs infected with 10 moi of HSV-1 for 16 h (hpi). gD positivity represents viral infection, Hoechst 33334 stains nuclear DNA. (B) The number of gD positive cells in (A) were determined by counting 10 different fields under the fluorescent microscope. The data represent the means of 3 independent stainings (counting 10 fields each) ± SEM. The p values are the following: HSV-1 versus mock, p < 0.001 for 24 and 48 h; HSV-1-infected Bax/Bak-/- versus HSV-1-infected WT cells: p = 0.01 for 24 h, p = 0.05 for 48 h, p < 0.001 for 72 h, n = 5. (C) Viral titers determined by the plaque assay and depicted as Log10 Plaque Forming Units (PFU)/ml after infecting U937 vector control (pMEP) and Bcl-2-overexpressing (Bcl-2) monocytes with 50 moi or infecting SV40 TAg WT and Bax/Bak-/- MEFs with 10 moi of HSV-1 for up to 72 h. Data are the means of at least three independent experiments ± SEM. The p values are < 0.001 for U937 Bcl-2 versus pMEP and SV40 TAg Bax/Bak-/- versus WT at 48 and 72 hpi, n = 4. (TIF) [file pone.0126645.s002.tif]

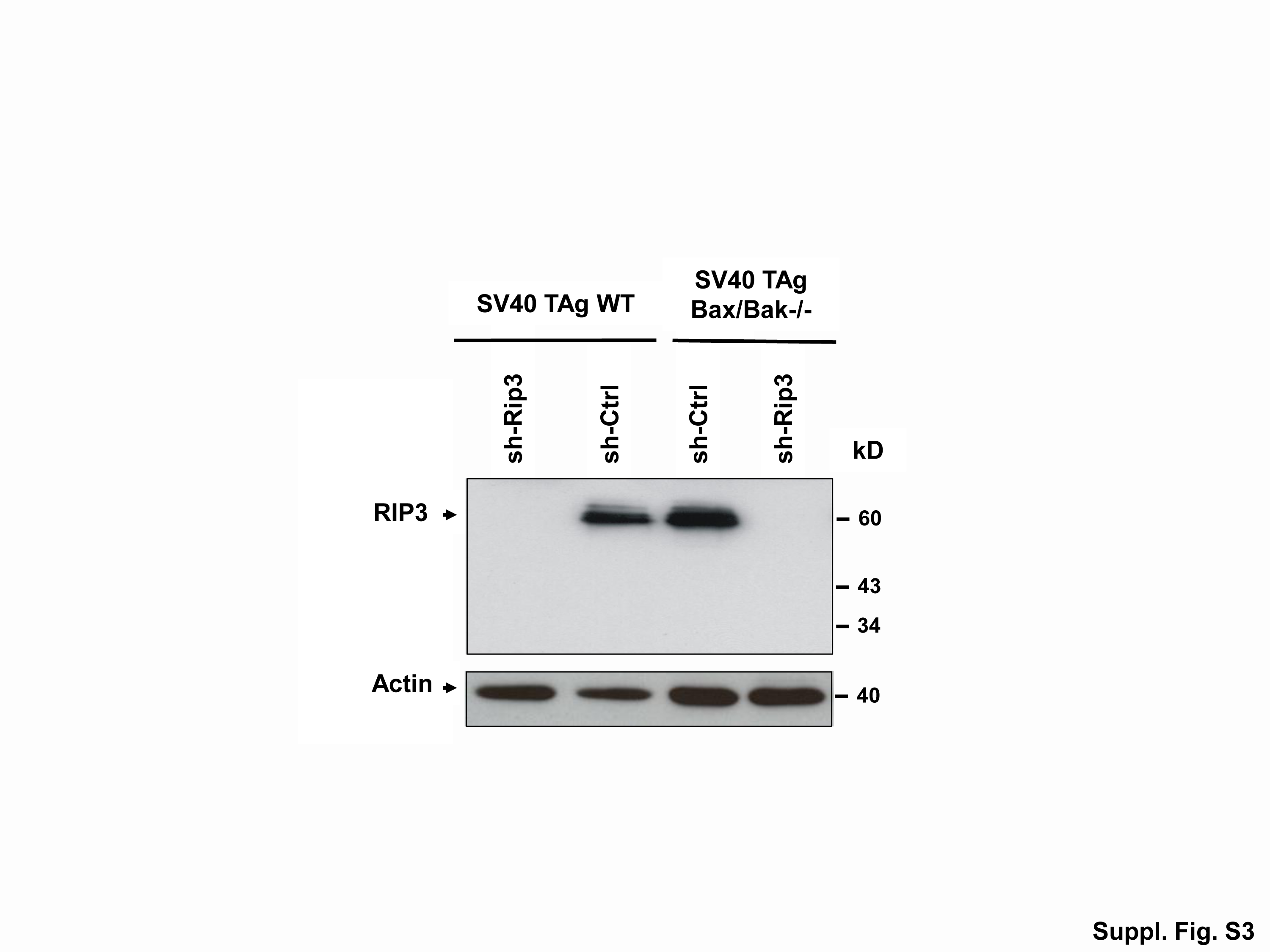

Supplement: S3 Fig — Anti-RIP3 western blot analysis of total extracts from mixed populations of puromycin-selected, SV40 TAg WT and Bax/Bak-/- MEFs infected with lentiviruses carrying a scrambled shRNA (sh-Ctrl) or an shRNAs for mouse RIP3 (sh-Rip3). Anti-actin as loading control. (TIF) [file pone.0126645.s003.tif]

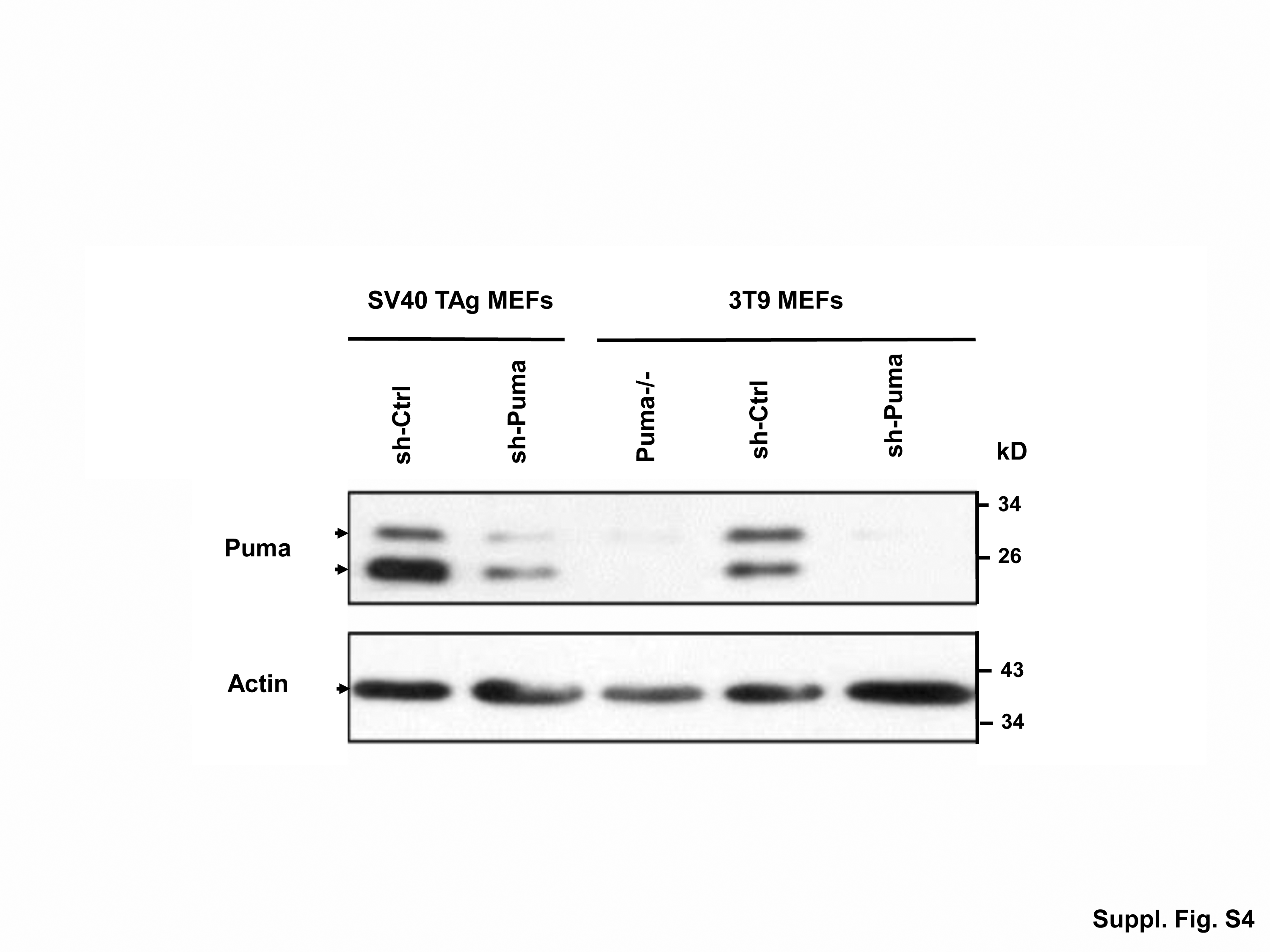

Supplement: S4 Fig — Anti-Puma western blot analysis of total extracts from mixed populations of puromycin-selected, SV40 TAg-transformed and 3T9-immortalized MEFs infected with lentiviruses carrying a scrambled shRNA (sh-Ctrl) or shRNAs for mouse Puma (Sigma Open Labs). For comparison, an extract from 3T9 Puma-/- MEFs is shown. Anti-actin as loading control. (TIF) [file pone.0126645.s004.tif]

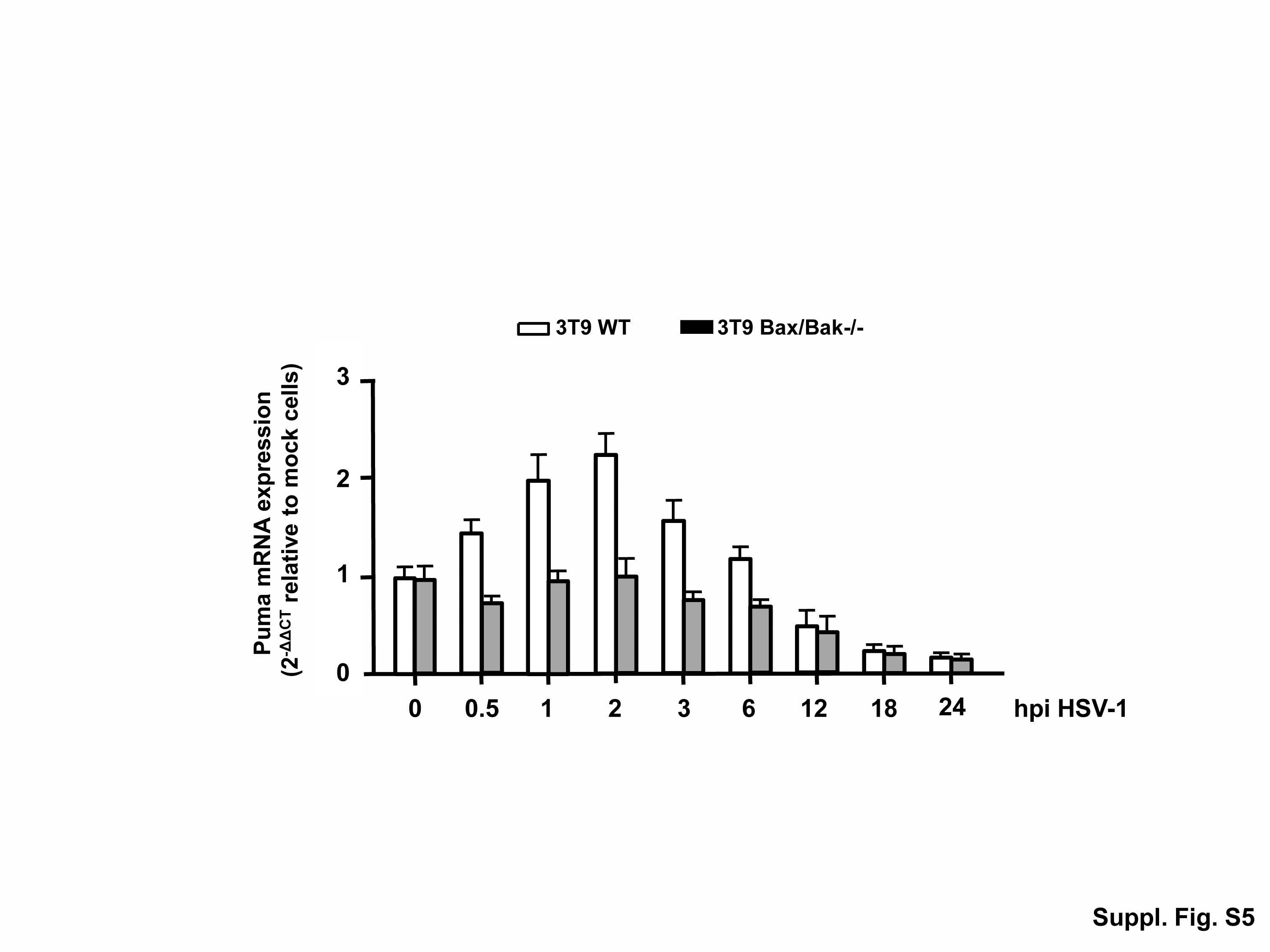

Supplement: S5 Fig — Quantitative/real time reverse transcriptase PCR (qRT-PCR) of Puma mRNA isolated from 3T9-immortalized WT and Bax/Bak-/- MEFs infected with 10 moi of HSV-1 for 0, 0.5, 1, 2, 3, 6, 12, 18 or 24 h. The mRNA values were normalized to the ribosomal housekeeping S18 gene and depicted as 2-∆∆Ct relative to mock cells (see Materials and Methods for details). Data are the means of at least three independent experiments using three different clones of 3T9 WT and Bax/Bak-/- cells ± SEM. The p values are the following: HSV-1 versus untreated: p = 0.05 for 0.5 and 6 h, p = 0.01 for 1, 2 and 3 h, n = 3. (TIF) [file pone.0126645.s005.tif]

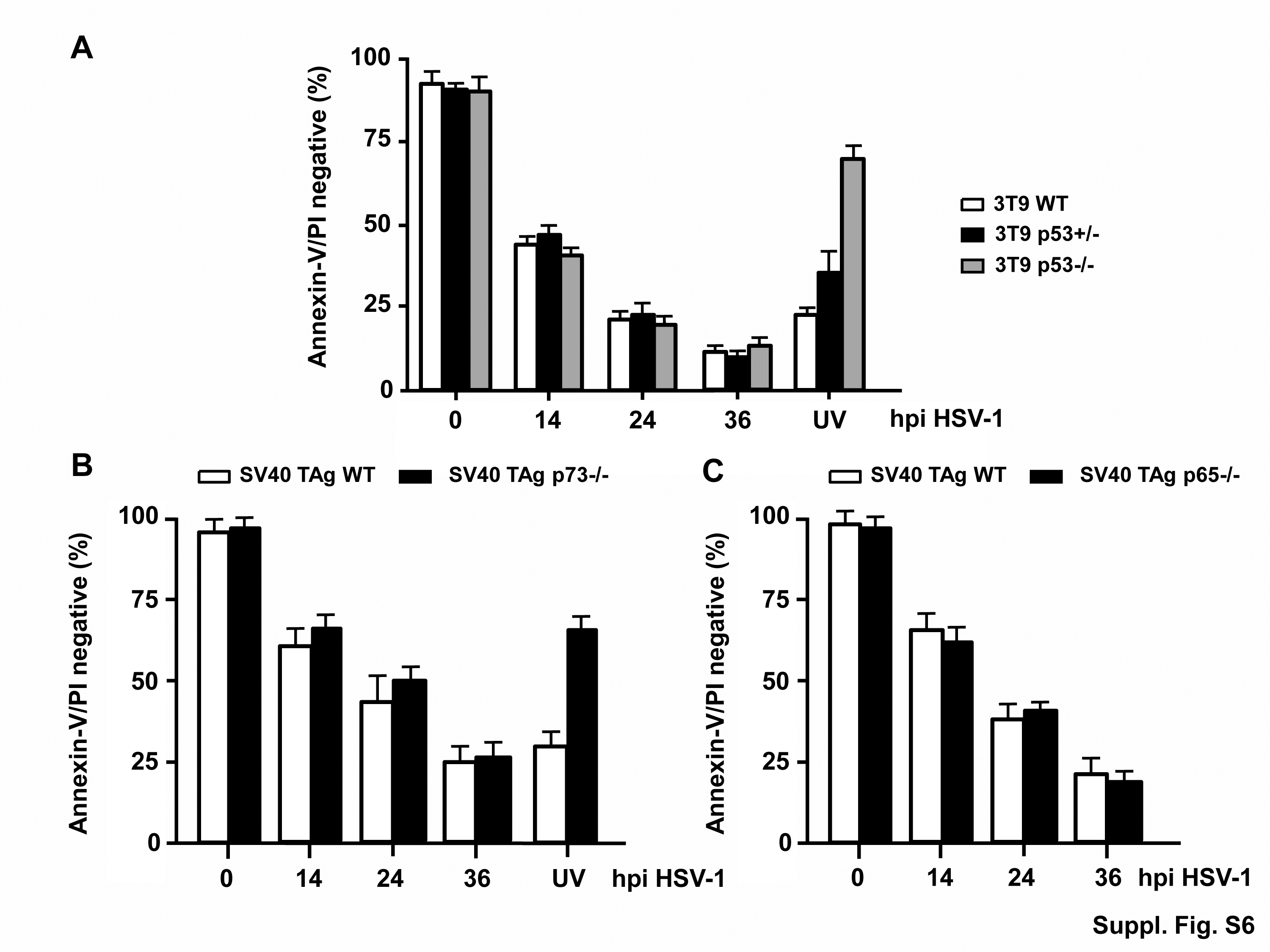

Supplement: S6 Fig — Annexin-V/PI FACS analysis of (A) 3T9-immortalized WT, p53-/+ and p53-/- MEFs, (B) SV40 TAg-transformed WT and p73-/- MEFs or (C) SV40 TAg-transformed WT and p65 NFκB-/- MEFs, infected with 10 moi of HSV-1 for 0, 14, 24 or 36 h (hpi). In (A) and (B) the cells were also exposed to UV light (100 J/m2) for 24 h as a positive control. Data are the means of at least three independent experiments using two different clones of WT and knock-out cells ± SEM. The p values are < 0.001 for UV-treated p53-/- versus WT and UV-treated p73-/- versus WT MEFs, n = 3. (TIF) [file pone.0126645.s006.tif]
